# Thermokarst landscape exhibits large nitrous oxide emissions in Alaska’s coastal polygonal tundra

**DOI:** 10.1038/s43247-024-01583-5

**Published:** 2024-08-30

**Authors:** Josh Hashemi, David A. Lipson, Kyle A. Arndt, Scott J. Davidson, Aram Kalhori, Kyle Lunneberg, Lona van Delden, Walter C. Oechel, Donatella Zona

**Affiliations:** 1 Biology Department, San Diego State University, San Diego, CA USA; 2 Department of Land, Air and Water Resources, University of California Davis, Davis, CA USA; 3 Alfred Wegener Institute Helmholtz Centre for Polar and Marine Research, Potsdam, Germany; 4 Woodwell Climate Research Center, Falmouth, MA USA; 5 University of Plymouth School of Geography, Earth and Environmental Sciences, Plymouth, UK; 6 GFZ German Research Centre for Geosciences, Potsdam, Germany; 7 Department of Geography, University of Exeter, Exeter, UK; 8 Department of Animal and Plant Sciences, University of Sheffield, Sheffield, UK

**Keywords:** Element cycles, Cryospheric science

## Abstract

Global atmospheric concentrations of nitrous oxide have been increasing over previous decades with emerging research suggesting the Arctic as a notable contributor. Thermokarst processes, increasing temperature, and changes in drainage can cause degradation of polygonal tundra landscape features resulting in elevated, well-drained, unvegetated soil surfaces that exhibit large nitrous oxide emissions. Here, we outline the magnitude and some of the dominant factors controlling variability in emissions for these thermokarst landscape features in the North Slope of Alaska. We measured strong nitrous oxide emissions during the growing season from unvegetated high centered polygons (median (mean) = 104.7 (187.7) µg N_2_O-N m^−2^ h^−1^), substantially higher than mean rates associated with Arctic tundra wetlands and of similar magnitude to unvegetated hotspots in peat plateaus and palsa mires. In the absence of vegetation, isotopic enrichment of ^15^N in these thermokarst features indicates a greater influence of microbial processes, (denitrification and nitrification) from barren soil. Findings reveal that the thermokarst features discussed here (~1.5% of the study area) are likely a notable source of nitrous oxide emissions, as inferred from chamber-based estimates. Growing season emissions, estimated at 16 (28) mg N_2_O-N ha^−1^ h^−1^, may be large enough to affect landscape-level greenhouse gas budgets.

## Abstract (web summary)

Growing season nitrous oxide emissions from thermokarst can be significant and may influence greenhouse gas budgets, according to flux measurements from the North Slope of Alaska.

## Introduction

Greenhouse gas (GHG) dynamics in permafrost ecosystems have been shifting due to increasing temperatures, active layer thickness, and hydrology with positive feedbacks on warming^1^. As permafrost soils make up one of the largest terrestrial reservoirs of carbon (C) and nitrogen (N)^2–4^, accumulated over millennia due to cold, water-saturated soils with slow decomposition^5^, increased attention has been given to GHG dynamics in permafrost regions over recent decades^6–13^. The majority of regional GHG studies have focused on C emissions (i.e., carbon dioxide (CO_2_) and methane (CH_4_)), outlining effects of, among others, seasonality^9,10,14,15^, landscape heterogeneity^16,17^, vegetation composition^18^ and vegetation density^19^. However, few studies have reported the flux dynamics of nitrous oxide (N_2_O), an ozone depleting substance and powerful GHG with a 100-year global warming potential (GWP_100_) 273 times that of CO_2_^20^.

N_2_O is produced from various biological and chemical processes happening simultaneously in the soil^21,22^. Production pathways of N_2_O are predominantly via nitrification, where N_2_O is a by-product in the oxidation of ammonium (NH_4_^+^) to nitrate (NO_3_^−^), and denitrification, where N_2_O is an intermediate in the reduction of nitrite (NO_2_^−^) and NO_3_^−^ to produce dinitrogen (N_2_) gas^21^. These two processes are interconnected and mainly driven by temperature, oxygen availability, and substrate availability and are limited in high-latitude ecosystems with short growing seasons^21^. N_2_O emissions have therefore often been considered negligible in permafrost regions, because of limited mineral N availability due to cold and wet environmental conditions^21^. Warming can lead to increased decomposition, mineralization, and release of N, previously locked in organic matter rich, permafrost-dominated Arctic soils^21,23–25^. Additions of this released bioavailable N can then act as a substrate for increased N_2_O production.

Strong plant competition for available inorganic N can reduce the production and emission of N_2_O in vegetated areas^26^. Plants ultimately absorb most of the bioavailable N due to the greater N demand by plants compared to the supply^27,28^. High microorganism turnover (3–5 days) results in a redistribution of soil N while plants slowly accumulate large portions of available N due to a lower turnover (1–3 months)^28^. However, unvegetated areas, common in Arctic regions^29–32^, remove competition for N by vascular plants and can result in higher rates of N_2_O production due to increased inorganic N availability for nitrification and denitrification processes^33,34^.

Emissions of N_2_O can be difficult to capture due to their high spatial and temporal variability on the landscape scale as well as on the micro-scale. This is due, in part, to N_2_O production happening during both aerobic and anaerobic soil conditions, which can simultaneously occur on the microscale in the highly complex soil matrix^35^. With increasing water filled pore space (WFPS), N_2_O production can shift from nitrification to incomplete denitrification, provided there is adequate NO_3_^−^ available, and eventually ends in N_2_ release when the soil is completely water saturated, limiting oxygen availability^22^. These dynamic processes contribute to the difficulty in upscaling N_2_O budgets, particularly in remote Arctic regions, as data collection campaigns may be sparse. Despite this, some research suggests that N_2_O emissions from permafrost ecosystems may have a significant and growing impact on the global N_2_O budget, contributing 0.14–1.27 Tg N_2_O-N per year (7% of global budget)^21^. Increasing soil temperatures and associated hydrological changes may facilitate conditions favorable for increased N cycling^36^. Given this potential positive feedback on warming, a better understanding of the response of N_2_O dynamics to warming and associated environmental changes in permafrost regions is needed.

Despite the number of studies on GHG fluxes, few in-situ N_2_O measurements have been published from the North Slope of Alaska^21^. The North Slope of Alaska is comprised of a patchwork of landscape features that includes lakes, ponds, drained lake basins, drained upland tundra and polygonal tundra with high levels of organic C and N^37–39^. Polygonal tundra are characterized by surface relief created by the common development and growth of ice wedges. Over time, these ice wedges lift areas of the soil, creating ridges and forming complex wetlands with distinct polygonal patterns that vary in position of the water table^40^.

Landscape heterogeneity in this region is due, in part, to freeze-thaw dynamics^40^. In particular, polygonal tundra, extending over an estimated 65% of the Arctic Coastal Plain^37^ and covering 3% of Arctic landmass (~250,000 km^2^)^41^, can result in significant variability in vegetation composition^42,43^, hydrology^38,40,44,45^, GHG dynamics^38,46,47^, and a wide range of GHG budget estimates^10,17,48,49^. Complex interactions of hydrology, ice wedge dynamics, and freeze-thaw cycles result in high-centered polygons, i.e., soil mounds that protrude above the water table^40,50^. Cryoturbation, thaw processes, thermal erosion, and changes in hydrology can destabilize and shift overlying soil structures^51,52^ disrupting the rooting structures of vascular plants, and causing high-centered polygons to degrade^53^. This can result in thermokarst-affected high-centered polygon features with exposed and unvegetated soil (hereafter referred to as “thermokarst polygons”) that can increase the rate of mineralization of N and affect plant-microbe competition for inorganic N^21,54^. Notably, the complex feature mosaic of the North Slope of Alaska landscape has been identified to have a high N_2_O potential from airborne eddy covariance screenings^55^ with the source feature remaining unknown.

The aim of this study was to identify the role of progressive thermokarst development and thermal erosion on N_2_O emissions with the expectation that areas with little or no vegetation within polygonal tundra of the North Slope of Alaska exhibit similarly high levels of N_2_O emission, comparable to the previously identified peat circle (Russia)^29^ and palsa (Finland)^30^ hotspots. Further, we address whether these polygon features have a larger climate forcing potential than previously assumed, when accounting for the much larger warming power of N_2_O compared to CO_2_^20^. Our estimates of chamber based N_2_O emissions, in conjunction with previous airborne eddy covariance measurements^55^ indicate that these thermokarst polygons and resulting bare spots may be intensive enough to affect the GHG budget on the landscape scale of the North Slope of Alaska.

## Results and discussion

### In-situ GHG flux measurements

**Fig. 1:**
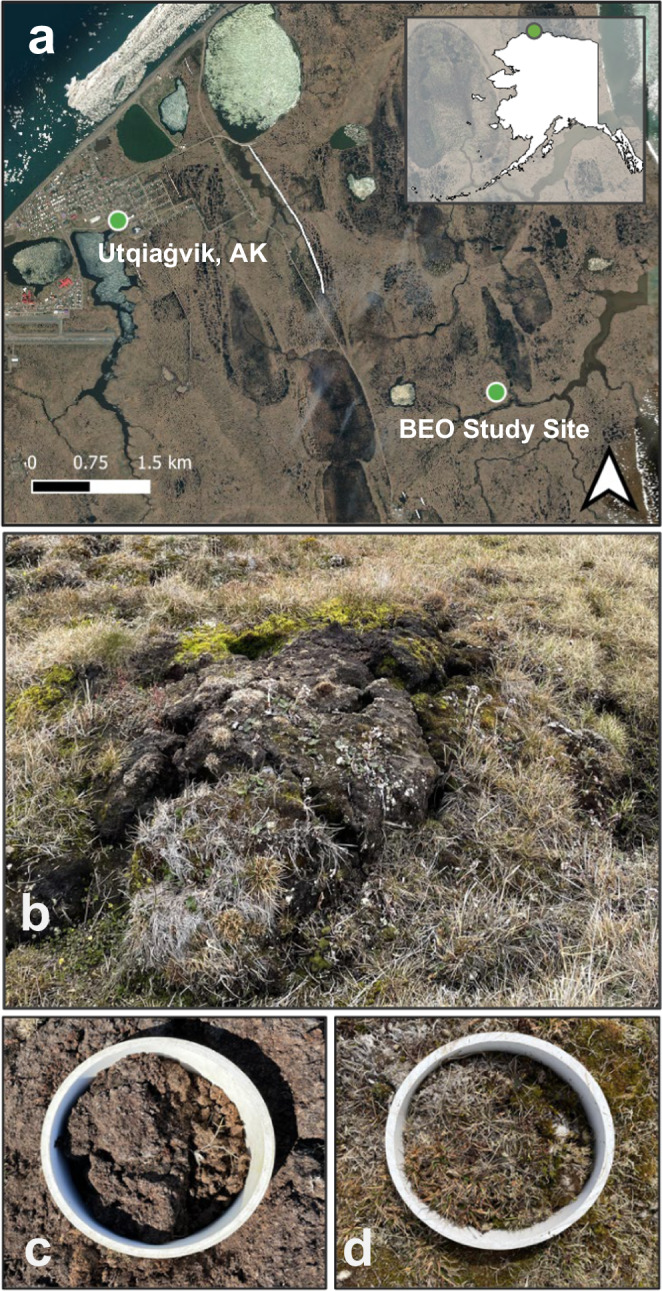
Overview of the site location and studied landscape features. **a** Study area near Utqiaġvik, AK and (**b**) eroding high center polygonal landscape feature with photos of collars with (**c**) unvegetated and (**d**) vegetated surface. Map (**a**) source credits: Esri, Maxar, GeoEye, Earthstar Geographics, CNES/Airbus DS, USDA, USGS, AeroGRID, IGN and the GIS User Community.

GHG fluxes were estimated using the static chamber technique on the Barrow Environmental Observatory (BEO), a polygonal tundra south of Utqiaġvik, Alaska (Fig. 1a). We report fluxes of both N_2_O and CO_2_ (Net Ecosystem Exchange (NEE)) to show the combined climate forcing potential of two of the main GHGs in these high latitude systems. Measurements were taken at thermokarst polygon surfaces (Fig. 1b; Supplementary Fig. 1) in unvegetated (Fig. 1c), and nearby vegetated areas (Fig. 1d) during the growing season (July). Vegetated and unvegetated features experienced a similar range of water table, soil water content, soil temperature, and thaw depth (Supplementary Fig. 2). Stable isotope and carbon to nitrogen (C:N) ratios were measured to support the interpretation of GHG flux dynamics by site and soil depth relating to the influence of the presence of vegetation cover.

**Fig. 2:**
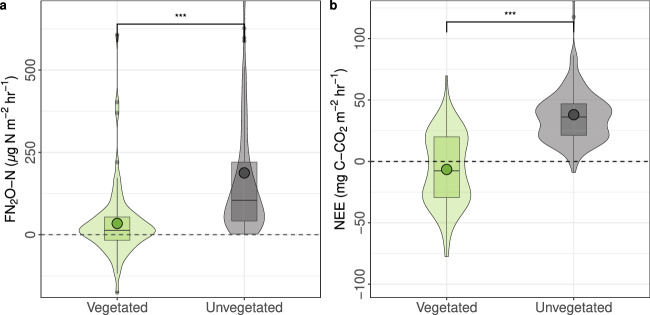
Nitrous oxide and carbon dioxide fluxes at vegetated and unvegetated areas. Comparison of (**a**) FN_2_O-N (μg N m^−2^ h^−1^) and (**b**) FC-CO_2_ (NEE) (mg C m^−2^ h^−1^) at areas with vegetated and unvegetated soil surfaces. Circles represent means and violin plots indicate the distribution of data. *n* = 182 at unvegetated sites *n* = 80 at vegetated sites. Asterisks indicate significance value: *** = *p* < 0.001 (ANOVA). Some positive outliers in unvegetated areas were not included in figures for better graphical representation.

Unvegetated areas (number of measurement locations = 20) on thermokarst polygons show significantly higher (*p* < 0.001) emissions of N_2_O (median (mean ± standard error) = 104.7 (187.7 ± 17.4) µg N_2_O-N m^−2^ h^−1^) in comparison with vegetated areas (number of measurement locations = 10; 13.5 (34.2 ± 12.1) µg N_2_O-N m^−2^ h^−1^) (Fig. 2a). The emissions from unvegetated areas reported here are more than two orders of magnitude higher than the median (mean) rate associated with permafrost wetlands (0.8 (5.2) µg N_2_O-N m^−2^ h^−1^) and substantially higher than emissions measured from Arctic peatlands and upland tundra (2.5 (24.8) & 1.4 (8.8) µg N_2_O -N m^−2^ h^−1^, respectively)^21^. Emissions are also higher than those reported from unvegetated areas in permafrost regions in general (18 (42) µg N_2_O-N m^−2^ h^−1^)^21^, and close to mean emissions found from previously identified Arctic N_2_O hotspots from unvegetated peat circles (~230 µg N_2_O -N m^−2^ h^−1^)^29^ and palsa mires (~270 µg N N_2_O -N m^−2^ h^−1^)^30^. N_2_O emission rates from unvegetated surfaces of thermokarst polygons are comparable to mean tropical organic soils (up to 125 µg N_2_O-N m^−2^ h^−1^)^56^, highlighting the importance of permafrost regions and Arctic tundra in the global N_2_O cycle. Analysis of UAV imagery across the study area reveals that approximately ~1.5% of the land surface consists of these unvegetated features (Supplementary Fig. 3). Based on this data, area-adjusted estimates suggest midday N_2_O-N emissions are around 16 (28) mg ha^−1^ h^−1^ during the growing season. Although emissions from barren regions reported here do not account for the full diurnal cycle (measurements took place between 9:00 and 16:00), clear partial diurnal trends are observed (Supplementary Fig. 4) illustrating the importance of diurnal fluctuations for N_2_O emissions estimates.

Emission rates of N_2_O at vegetated areas on thermokarst polygons are also slightly higher in comparison to the above estimates for permafrost wetlands. High-centered polygons are better drained than surrounding lower areas that are commonly inundated. Therefore, these features are better aerated and have higher oxygen availability than the surrounding permafrost wetlands. The greater oxygen concentrations and higher decomposition would be expected to result in greater N availability and N_2_O production via nitrification. Cryoturbation and freeze-thaw cycles in high-centered polygons may also mix N from deeper soil layers near or at the permafrost table. This can mobilize existing pockets of N_2_O and inorganic nitrogen within the permafrost, bringing them closer to the active layer for potential uptake thereby increasing mineral N availability for N_2_O production^23,24,57,58^. In addition, vegetation communities on these structures contain moss and lichen communities that are associated with biological nitrogen fixation. This can increase the soil inorganic N pool^59,60^ and possibly - through increased mineral N availability - N_2_O production, relative to inundated areas^22^.

Measurements of NEE reveal significantly larger CO_2_ emissions from unvegetated surfaces (*p* < 0.001, DF = 28), showing these areas to be a source median (mean ± standard error) = (36.2 (38.0 ± 1.88) mg C-CO_2_ m^−2^ h^−1^) compared to a weak sink at adjacent vegetated areas (−7.7 (−6.6 ± 0.08) mg C-CO_2_ m^−2^ h^−1^) during the daytime (Fig. 2b). The difference is likely primarily due to the absence of CO_2_ uptake through photosynthesis at the unvegetated surfaces, resulting solely in ecosystem respiration (ER). Fluxes of CO_2_ from vegetated and unvegetated areas are similar to previous estimates of NEE^61^ and ER^62^ respectively. When estimating the GWP_100_ of thermokarst polygons, the combined effect of CO_2_ and N_2_O nearly doubled the climate forcing potential (64.58 (89.07 ± 6.6) mg CO_2_eq m^−2^ h^−1^) when compared to that of CO_2_ alone. This comparison only reflects midday conditions and does not take the diurnal variability of both CO_2_ and N_2_O into account. It is possible that the relative effect of CO_2_ emissions would be higher if diurnal variability were considered, though the variability in N_2_O diurnals is unknown.

### Variability in and controls on N_2_O emission strength

**Fig. 3:**
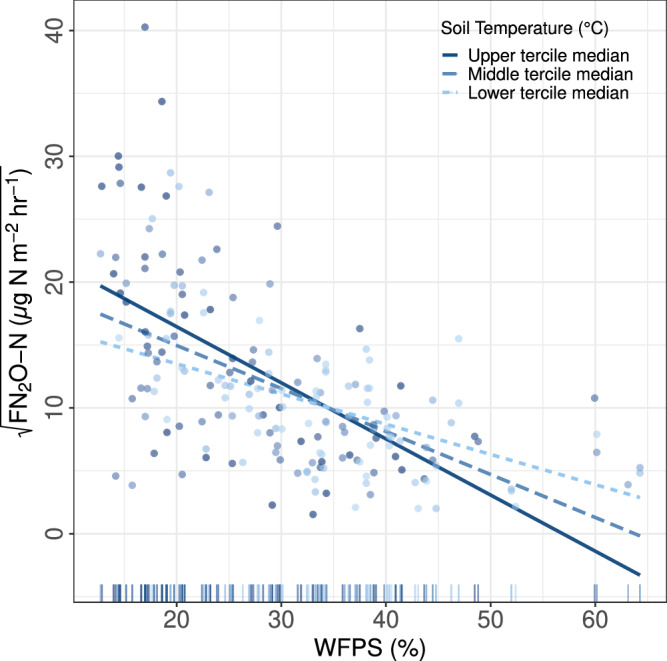
WFPS and soil temperature controls on N_2_O fluxes at unvegetated areas. The impact of the Interaction between WFPS (%) and soil temperature (°C) on FN_2_O (µg N m^−2^ h^−1^). Regression lines show a variable effect of WFPS on FN_2_O at the lower, middle upper tercile median. FN_2_O has been square root transformed to meet the normality and homoscedasticity assumptions required for analyses. The marginal rug plot above the x-axis shows the predictor relationship.

Linear mixed effects model output indicates that higher N_2_O emissions at unvegetated areas are associated with lower WFPS and tend toward higher temperatures (Fig. 3, Supplementary Table 1). No relationships between ancillary data and N_2_O fluxes were found to be significant in vegetated areas. The difference in patterns of emissions among vegetated areas and unvegetated areas is likely due, in part, to reduced competition for inorganic N by vascular plants, relatively warm soil temperatures and increased oxygen availability due to greater soil aeration. Bulk density was significantly lower in unvegetated soil (0.073 ± 0.004 g cm^−3^) than in vegetated areas (0.119 ± 0.006 g cm^−3^), possibly allowing for increased oxygen penetration into the soil column and thus, conditions more favorable for nitrification, at least in the upper 15 cm where reported soil water content was measured. N_2_O emissions peaked at around 20% WFPS in the top 15 cm (Fig. 3). While this may provide support for nitrification-based N_2_O production, denitrification has been identified as a dominant N_2_O emission pathway for tundra regions^34^ and barren mineral polygon tundra^63^, and thus likely occurs at deeper soil layers (>15 cm) that have increased soil water content and limited oxygen availability. The significant interaction of WFPS and soil temperature (*p* = 0.02) in the highest performing model (pseudo R² (fixed effects) = 0.34) may give some indication of this as well, as higher surface temperatures with lower WFPS could be correlated with warmer conditions in deeper soil promoting enhanced denitrification. Though there is an obvious influence of temperature over the microbial processes governing N_2_O emissions, in permafrost regions, N_2_O emissions have been found to be dominantly controlled by substrate availability and conditions associated with oxygen availability, such as soil moisture and soil pore size^64^. WFPS is tightly related to soil redox potential and oxygen availability, as soil diffusivity increases with lower bulk density and lower soil water content^22^. Thaw depth showed no correlation (Supplementary Fig. 5) and decreased multivariate model performance (Supplementary Table 1). Stronger correlations of N_2_O emissions with thaw depth would be expected with permafrost thaw due to the introduction of new organic matter rather than seasonally thawing active layer^65^.

### Carbon and Nitrogen composition of soil environment

**Fig. 4:**
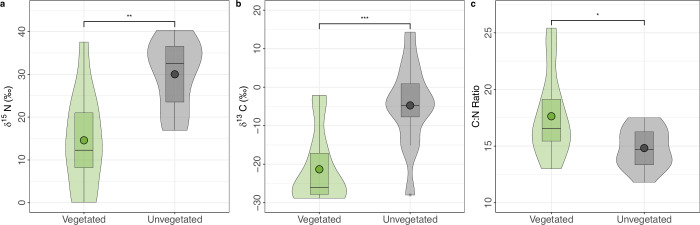
δ ^15^N, δ ^13^C and C:N ratio. Comparison of (**a**) δ 15 N (‰), (**b**) δ 13 C (‰), and (**c**) C:N ratio from soil samples with unvegetated and vegetated surface. Circles represent means and violin plots indicate the distribution of data. Asterisks indicate significance level: *p < 0.01; **p < 0.005; ***p < 0.001 (Two sample unpaired t-test).

Soil samples from areas with no vegetation were significantly higher in both δ ^15^N (30 ± 2.34‰) and δ ^13^C (−4.74 ± 3.2‰) content than in vegetated soils (δ ^15^N: 14.58 ± 3.3‰; δ ^13^C: = −21.32 ± 2.8‰) (Fig. 4a, b). The elevated δ ^13^C signature of unvegetated soils may be due to (1) the influence of microbial products derived from older labile plant compounds, combined with the preferential loss of lighter carbon over time and a lack of seasonal inputs, (2) localized carbonate accumulation or (3) a combination of these two processes. Isotopic enrichment of ^15^N in unvegetated soil areas indicates a larger loss of gaseous N species via microbial processes such as nitrification and denitrification, as plant uptake does not occur^34,66^. Ammonium volatilization is likely limited due to the acidic soils in this region^67^, however may occur to some extent if unvegetated areas exhibit localized increased alkalinity. Both nitrification and denitrification are highly sensitive to changes in oxygen availability and due to the variable nature of hydrology in polygonal tundra^40,68^, microsite variability in moisture content may support high rates of N_2_O production through both of these pathways simultaneously. It is possible that emissions of N_2_O from thermokarst polygons were primarily from nitrification due to the strong relationship with properties governing oxygen availability. However, at deeper, more saturated soil levels closer to the permafrost table, oxygen availability is likely more limited and could allow for denitrification or nitrifier denitrification to substantially contribute to surface flux^34^. Particularly noteworthy are transition zones, where abrupt changes in oxygen availability occur. These transition zones may create conditions suitable for simultaneous denitrification and nitrification, and contribute to elevated N_2_O emissions from these specific micro-environments. Deeper areas, nearer to the permafrost table could also contribute the overall N_2_O emissions through available NO_3_^−^ and/or NO_2_^−^ release directly from the permafrost.

Data from δ ^15^N and δ ^13^C in unvegetated areas showed no significant relationship with depth in the top 15 cm of the soil column (Supplementary Fig. 6) though the deepest soil layer in vegetated areas had elevated δ ^13^C, possibly indicated some level of freeze thaw mixing with nearby unvegetated soils. Although mean values for δ^15^N (~33‰) were higher at deeper areas compared to shallower depths (~29‰), these differences lacked significance due to the broad ranges observed at shallower depths. This may suggest a correlation with soil layers deeper than those measured here, potentially supporting increased denitrification in deeper soil layers. Although previous research has demonstrated a relatively uniform distribution of sequences corresponding to denitrification respiratory pathway genes with soil depth in this locale^69^, nitrate profile analysis from polygon rims show low nitrate at the deepest soil layers, as nitrate is rapidly reduced in these suboxic environments^70^. The high variability of these data likely reflects that the pathways of N loss leading to isotopic enrichment are episodic and therefore highly variable.

Significantly lower C:N ratios (*p* = 0.03) were found in unvegetated soils (14.82 ± 0.52) than in vegetated (17.63 ± 1.1) (Fig. 4c). This potentially provides support of greater N bioavailability for N_2_O production in unvegetated soils^71^. Differences in C:N ratio were driven by a higher N content in unvegetated areas (17.6 ± 0.7 mg N g^−1^) than in vegetated soils (10.3 ± 1.4 mg N g^−1^) (Supplementary Fig. 7). Total C and N content per volume was not significantly different when standardizing with mean bulk density measurements (Supplementary Fig. 7). In vegetated soil areas, C:N ratios generally decreased with depth in the top 15 cm of the soil column (Supplementary Fig. 8), likely related to plant N uptake occurring at increased rates closer to the surface where plant root tissue is more abundant^26,27^. Mean and median total C and N were both higher at deeper soil depth in vegetated areas, albeit not significantly (Supplementary Fig. 9).

### Implications of N_2_O emissions on the landscape scale

Data presented here have important implications for regional estimations of future N_2_O emissions due to substantial hydrological, thermal, active layer, and land surface changes expected in high latitude ecosystems in coming decades. Arctic wetlands maintain water tables near or above the soil surface for most or all of the year, due to limited drainage created by the permafrost barrier, facilitating anaerobic conditions in the soil column^67^. However, as regional warming continues, permafrost degradation could cause increased active layer depths, lateral movement of water and drainage of polygonal tundra^40,72^. These processes heighten the likelihood of barren soil exposure due to thermokarst and thermal erosion, evident in a sixty-fold surge in the development and expansion of retrogressive thaw slump thermokarst features in recent decades^73^. This expansion, coupled with surface disturbances, amplifies the potential for Arctic wetlands to emerge as globally significant sources of N_2_O emissions. Additional N_2_O flux measurements across a wide range of sites in heterogeneous tundra environments are needed to understand and document the variability of emissions due to topography, vegetation and environmental conditions. High-emitting landscape features like thermokarst polygons may substantially increase with likely changes in temperature, hydrology, and active layer depth^40^.

Previous assumptions of negligible N_2_O emissions rates from Arctic environments are increasingly challenged based on low, but evident circumarctic emissions around 1.25 µg N_2_O-N m^−2^ h^−1^ ^21^ with an increasing body of evidence of high emission features^29,30,74^ emitting up to >260 µg N_2_O-N m^−2^ h^−1 57^. Though low N_2_O emissions or N_2_O uptake driven by denitrification is often reported in high latitude wetlands^21^, high landscape scale, growing season N_2_O emissions from the North Slope of Alaska were identified using aircraft eddy covariance, showing a mean of ~99 µg N_2_O-N m^−2^ h^−1^ ^55^. The results presented here identify a possible contributing source of these landscape relevant N_2_O emissions, highlighting the importance of small-scale landscape features (≤0.5 m^2^ area). The larger distribution of thermokarst polygons across the North Slope region is unknown, primarily attributable to the challenges posed by their small size, rendering them less discernible through satellite imagery.

UAV imagery over a limited area encompassing the study region places estimates of the feature coverage at ~1.5% of the land surface, though how representative this estimate is for the North Slope region and polygonized tundra in general remains uncertain. The mean flux rate adjusted for the estimated feature coverage is 28 mg N_2_O-N ha^−1^ h^−1^. This is still significantly lower than those estimated from airborne eddy covariance. The potential application of larger scale UAV imagery orthomosaics could help identify a more constrained distribution of these features and provide a means for regional upscaling of fluxes to compare emissions reported here with the previously mentioned estimates from airborne eddy covariance. Persistent disparities between these estimates may signify the existence of stronger N_2_O emissions from thermokarst polygons not captured in the presented data. This also indicates further unaccounted-for high-emitting landscape features, such as boundary layers to water bodies or certain topography features that could result in additional N_2_O hotspots.

While the data presented here offer insight into a novel N_2_O source, there are several limitations and avenues for future research. In particular, data are needed on soil composition, nitrogen cycling, microbial community composition, and inorganic nitrogen content across soil depths to elucidate elevated δ^13^C, dominant N_2_O production pathways, and identify zones of N_2_O production. High resolution imagery over larger spatial extents are needed to improve scaling efforts as the more widely available coarser resolution imagery are unable to detect the sub-meter features discussed here. There is currently a paucity of in-situ data over longer time periods making upscaling to regional estimates very challenging. As conditions favorable for N_2_O production and release can rapidly change^75^, the emission rates observed here are only representative of midday growing season emissions.

The annual contribution to the global N_2_O budget from these regions is still currently unknown. In particular, measurements of N_2_O flux data from outside of the growing season are lacking. Year-round flux measurements at the landscape level, e.g., eddy covariance and automated chamber systems are needed to determine diurnal behavior and seasonal budget dynamics and to better inform model parameterizations. Arctic wetlands exhibit strong emissions of both CO_2_ and CH_4_ during seasonal shoulder periods, notably in the autumn zero curtain period of soil freezing^9,10,76^. As plant uptake of inorganic N should be limited outside of the growing season due to lower plant productivity and plant senescence, significant emissions of N_2_O may occur during this period. Likewise, data collected during the spring could reveal large budgetary contributions as the spring thaw period has been associated with peak N_2_O emission^32^. A budgetary understanding of regional emissions of N_2_O will likely increase warming potential estimates of Arctic wetlands and account for an additional warming feedback. The magnitudes of the emissions from these thermokarst polygon features highlight the importance and further need of in-situ N_2_O source and sink identification for future climate forcing potential from warming Arctic environments.

## Materials and methods

### Study site

This study was conducted near Utqiaġvik, Alaska in a well-developed polygonal tundra consisting of high and low-center polygons, on the Barrow Ecological Observatory (BEO; 71 16’ 51”N, 156 26’ 44”W) (Fig. 1a). The BEO is on the Arctic Coastal Plain on the North Slope of Alaska and is predominantly (65%) polygonal tundra with the remainder of the landscape comprised of a combination of lakes, drained lake basins and upland tundra^37^. Soils in the BEO are in the continuous permafrost zone and are gelisols turbels (cryoturbated soils: 71–77%; orthels (mineral): 8%; organic soils: 1%) with high levels total organic C and N (18% & 0.7% respectively)^18,77^. Vegetation primarily consists of wet sedges (*Carex aquatilis)* and mosses (*Sphagnum* spp. and *Drepanocladus* spp.) in heavily inundated areas such as low center polygons and troughs, and moss/lichen (*Polytrichum* spp. & *Dicranum* spp.) dominated communities in high-center polygon and ridge areas^43^. The water table is variable depending on landscape relief and can be as high as ≥20 cm above the ground surface and as low as ≥50 cm below the ground surface. Collar locations were only in thermokarst polygons with unvegetated soil or adjacent vegetated areas also on thermokarst polygons. All thermokarst polygon features had a water table at or below the active layer depth throughout the study period. Mean maximum active layer thaw depth in these features was estimated at ~40 cm.

### GHG flux and ancillary measurements

Static chamber fluxes were measured with a Gasmet GT5000 Terra Fourier transform infrared (FTIR) GHG analyzer and a clear, cylindrical polycarbonate chamber (50 cm height and 20 cm diameter) (Supplementary Fig. 10) in a closed system at a 1 Hz sampling rate. Due to the small chamber dimensions and low temperature variability at the sampling location, no pressure vent, cooling system or fan was added based on previously established chamber designs^18,43^, and relying on the pump of the GT5000 Terra to create adequate mixing within the chamber. The GT5000 Terra is capable of measuring multiple gases simultaneously by scanning the full infrared spectrum and calculating the concentrations of each gas in the sample based on its absorption with a precision of ± 3% and a minimum detectable concentration difference of 5 ppm and 7 ppb for CO_2_ and N_2_O, respectively^78,79^. FTIR enables the identification of unique regions with distinct peaks and characteristics within the measurement spectrum, effectively mitigating any issues related to measured gas cross-interference. Zero-point calibration was performed with N2 immediately before and after each use to ensure that any background signals or offsets in the values reported by the GT5000 Terra were minimized. Chamber collars were made of PVC (15 cm height and 20 cm diameter) and installed 3 days prior to GHG measurements at a depth of 10 cm. The chamber was ventilated prior to every measurement and placed on top of the collar ensuring an airtight connection via a rubber seal fitting the chamber to the collar (Supplementary Fig. 10). Following chamber placement, measurements were recorded over 7 min to obtain a stable increase or decrease in GHG concentration. Fluxes were calculated according to the linear slope fitting technique^80^ using linear regression to identify the change in concentration in the chamber headspace, including collar volume, over time and quality controlled by visual inspection. Fluxes were measured at 30 locations – 10 vegetated replicates and 20 unvegetated soil replicates – whenever weather permitted during July 2021 for a total of 263 measurements (8–10 measurements per collar). Measurements took place between 9:00 and 16:00 and the order was changed every day to ensure adequate temperature variation. The thermokarst polygon estimated coverage of 1.53% was based on UAV imagery of the study site (~5000 m^2^; Supplementary Fig. 3). The UAV imagery was collected using a DJIP4 Multispectral drone. Surveys were flown at 12 m above-ground level resulting in a sampling resolution of 0.9 cm/pixel. On-the-ground accuracy was maintained to less than 1 cm, using a connected Realtime kinetic base station^81^. Post-processing relied on Pix4Dmapper. Unvegetated regions were digitized in QGIS software (Open Source Geospatial Foundation) using measurement locations as reference data. The estimated coverage was calculated as the ratio digitized area of unvegetated features to the area of the UAV imagery extent.

Ancillary measurements included soil surface temperature, bulk density, thaw depth, soil water content, stable isotope ratios, and C:N ratios. Soil water content, soil surface temperature, and thaw depth were measured at the time of each chamber measurement at the flux collar throughout the study period (*n* = 263). Soil measurements and samples were taken from the top 15 cm of the soil column, separated into three 5 cm layers. Soil water content was measured with a 300 TDR soil moisture meter (Fieldscout, USA) over the top 15 cm from the soil surface. The conditions during summer 2021 were within the ranges reported by the long-term mean^82^, further supporting the representativeness of these measurements for emission rates. Soil surface temperature was measured with an TP7 infrared thermometer (Trotec, Germany). Thaw depth was measured with a small diameter metal rod inserted into the soil column until encountering resistance from the permafrost table. Bulk density was measured from soil samples from the top 15 cm of the soil column, collected at each collar location at the end of the study period, for a total of 30 samples. Soil samples were dried for 24 h at 60 °C in a drying oven and results expressed as dry weight per unit volume. WFPS was calculated by integrating bulk density, which represented the overall soil mass, and soil water content, indicating the water proportion according to ref. ^83^.

### Stable isotope analysis

Soil samples from the top 15 cm of the soil column were removed at both vegetated and unvegetated areas near where fluxes were measured using a handheld soil sampling corer (7 cm diameter, 15 cm height) at the end of the experiment. Soil samples consisted of four profiles with three depths (0–5 cm, 5–10 cm, & 10–15 cm) for a total of 24 samples, which were sieved for root removal. Samples were frozen and shipped to San Diego State University for stable isotope analysis. Samples were then separated into 5 cm depth segments (to check relationship with depth) using a band saw, placed in a drying oven at 65 °C for 48 h, then homogenized with a vibratory ball mill. Carbonate was not removed prior to analysis however, we would not expect this to contribute significantly to total C in these acidic, organic rich soils^67,84^. The abundance of ^15^N, ^13^C, and C:N ratios were measured using a continuous flow isotope ratio mass spectrometer (IRMS, Delta V Advantage, Thermo Fisher Scientific). A laboratory standard (USGS41, L-glutamic acid) was used as a reference material for the calibration of stable C and N measurements. Isotope values are reported in standard δ notation (‰) relative to Vienna PeeDee Belemnite (δ ^13^C) and air-N_2_ (δ ^15^N).

### Statistics and data analysis

All data analyses were performed in R software, version R 4.1.0^85^. Data organization was performed using the ‘data.table’ R package^86^. Repeated measures ANOVAs were used for site differences (unvegetated, vegetated) for both N_2_O and CO_2_ fluxes using collar location as a random variable to represent hierarchical structure, controlling for the pseudo replication related to measuring the same plots multiple times during the summer. A series of linear mixed effects models were used to predict the variability in FN_2_O. Model predictor variables included WFPS, soil temperature, thaw depth and various variable interactive terms. Linear mixed-effects model comparison showed that the models including soil temperature and WFPS as predictors for FN_2_O at unvegetated areas, along with an interactive term capturing their combined effect demonstrated superior predictive performance, as indicated by lower AIC relative to alternative models (Supplementary Table 1). N_2_O fluxes were square root transformed to meet the normality and homoscedasticity assumptions required for analyses. Assumptions of normality and homoscedasticity were verified with residual diagnostic tests. All model variables for were checked for multicollinearity (VIF < 2.3; Tolerance statistic >0.4) using the ‘olsrr’ R package^87^. Graphics were generated using the ‘ggplot2’^88^, ‘ggsignif’^89^, ‘cowplot’^90^, and ‘interactions’^91^ packages. Two sample unpaired t-tests were used for comparisons of stable isotope content and C:N Ratios (Fig. 4).

### Reporting summary

Further information on research design is available in the Nature Portfolio Reporting Summary linked to this article.

### Supplementary information

Transparent Peer Review file

Supplementary Material

Reporting Summary

## Data Availability

All data that support the findings of this study are openly available at: 10.5281/zenodo.8391857.

## References

[1] Natali, S. M. et al. Permafrost carbon feedbacks threaten global climate goals. *Proc. Natl Acad. Sci. USA* **118**, 1–3 (2021). 10.1073/pnas.2100163118 PMC8166174 34001617

[2] Hugelius, G. et al. Estimated stocks of circumpolar permafrost carbon with quantified uncertainty ranges and identified data gaps. *Biogeosciences* **11**, 6573–6593 (2014). 10.5194/bg-11-6573-2014

[3] Harden, J. W. et al. Field information links permafrost carbon to physical vulnerabilities of thawing. *Geophys. Res. Lett.* **39**, 1–6 (2012). 10.1029/2012GL051958

[4] Tamocai, C. et al. Soil organic carbon pools in the northern circumpolar permafrost region. *Glob. Biogeochem. Cycles* **23**, 1–11 (2009).

[5] Post, W. et al. Soil carbon pools and world life zones. *Nature* **298**, 156–159 (1982). 10.1038/298156a0

[6] Oechel, W. et al. Recent change of Arctic tundra ecosystems from a net carbon dioxide sink to a source. *Nature* **361**, 520–523 (1993). 10.1038/361520a0

[7] Oechel, W. et al. Transient nature of CO_2_ fertilization in Arctic tundra. *Nature* **371**, 500–503 (1994). 10.1038/371500a0

[8] Oechel, W. C. et al. Acclimation of ecosystem CO_2_ exchange in the Alaskan Arctic in response to decadal climate warming. *Nature* **406**, 978–981 (2000). 10984048 10.1038/35023137

[9] Oechel, W. C., Laskowski, C. A., Burba, G., Gioli, B. & Kalhori, A. A. M. Annual patterns and budget of CO_2_ flux in an Arctic tussock tundra ecosystem. *J. Geophys. Res.: Biogeosci.* **119**, 323–339 (2014). 10.1002/2013JG002431

[10] Zona, D. et al. Cold season emissions dominate the Arctic tundra methane budget. *Proc. Natl Acad. Sci. USA* **113**, 40–45 (2016). 26699476 10.1073/pnas.1516017113 PMC4711884

[11] Natali, S. M. et al. Large loss of CO_2_ in winter observed across the northern permafrost region. *Nat. Clim. Change* **9**, 852–857 (2019). 10.1038/s41558-019-0592-8 PMC8781060 35069807

[12] Bruhwiler, L. et al. The arctic carbon cycle and its response to changing climate. *Curr. Clim. Change Rep.* **7**, 14–34 (2021). 10.1007/s40641-020-00169-5

[13] Miner, K. R. et al. Permafrost carbon emissions in a changing Arctic. *Nat. Rev. Earth Environ.* **3**, 55–67 (2022). 10.1038/s43017-021-00230-3

[14] Commane, R. et al. Carbon dioxide sources from Alaska driven by increasing early winter respiration from Arctic tundra. *Proc. Natl Acad. Sci.* **114**, 5361–5366 (2017). 28484001 10.1073/pnas.1618567114 PMC5448179

[15] Taylor, M. A., Celis, G., Ledman, J. D., Bracho, R. & Schuur, E. A. G. Methane efflux measured by Eddy covariance in Alaskan Upland Tundra undergoing permafrost degradation. *J. Geophys. Res.: Biogeosci.* **123**, 2695–2710 (2018). 10.1029/2018JG004444

[16] Treat, C. C. et al. Tundra landscape heterogeneity, not interannual variability, controls the decadal regional carbon balance in the Western Russian Arctic. *Glob. Change Biol.* **24**, 5188–5204 (2018). 10.1111/gcb.14421 30101501

[17] Hashemi, J., Zona, D., Arndt, K. A., Kalhori, A., & Oechel, W. C. Seasonality buffers carbon budget variability across heterogeneous landscapes in Alaskan Arctic tundra. *Environ. Res. Lett.* **16**. 10.1088/1748-9326/abe2d1 (2021).

[18] Davidson, S. J. et al. Vegetation type dominates the spatial variability in CH_4_ emissions across multiple Arctic Tundra landscapes. *Ecosystems* **19**, 1116–1132 (2016). 10.1007/s10021-016-9991-0

[19] Andresen, C. G., Lara, M. J., Tweedie, C. E. & Lougheed, V. L. Rising plant-mediated methane emissions from arctic wetlands. *Glob. Change Biol.* **23**, 1128–1139 (2017). 10.1111/gcb.13469 27541438

[20] Forster, P. et al. The earth’s energy budget, climate feedbacks, and climate sensitivity. in *Climate Change 2021: The Physical Science Basis*. Contribution of Working Group I to the Sixth Assessment Report of the Intergovernmental Panel on Climate Change (eds Masson-Delmotte, V., Zhai, P., Pirani, A., Connors, S.L., Péan, C., Berger, S., Caud, N., Chen, Y., Goldfarb, L., Gomis, M.I., Huang, M., Leitzell, K., Lonnoy, E., Matthews, J.B.R., Maycock, T.K., Waterfield, T., Yelekçi, O., Yu, R. & Zhou, B.) (Cambridge University Press, Cambridge). 10.1017/9781009157896.009 (2021).

[21] Voigt, C. et al. Nitrous oxide emissions from permafrost-affected soils. *Nat. Rev. Earth Environ.* **1**, 420–434 (2020). 10.1038/s43017-020-0063-9

[22] Butterbach-Bahl, K., Baggs, E. M., Dannenmann, M., Kiese, R., & Zechmeister-Boltenstern, S. Nitrous oxide emissions from soils: how well do we understand the processes and their controls? *Philos. Trans. R. Soc. B: Biol. Sci.* **368**. 10.1098/rstb.2013.0122 (2013). 10.1098/rstb.2013.0122 PMC3682742 23713120

[23] Keuper, F. et al. A frozen feast: thawing permafrost increases plant-available nitrogen in subarctic peatlands. *Glob. Change Biol.* **18**, 1998–2007 (2012). 10.1111/j.1365-2486.2012.02663.x

[24] Beermann, F. et al. Permafrost Thaw and liberation of inorganic nitrogen in Eastern Siberia.*Permaf. Periglac. Process.* **28**, 605–618 (2017). 10.1002/ppp.1958

[25] Salazar, A., Rousk, K., Jónsdóttir, I. S., Bellenger, J.-P. & Andrésson, Ó. S. Faster nitrogen cycling and more fungal and root biomass in cold ecosystems under experimental warming: a meta-analysis. *Ecology* 101 10.1002/ecy.2938 (2020). 10.1002/ecy.2938 PMC7027553 31750541

[26] Subbarao, G. V. et al. Evidence for biological nitrification inhibition in Brachiaria pastures. *Proc. Natl Acad. Sci. USA* **106**, 17302–17307 (2009). 19805171 10.1073/pnas.0903694106 PMC2752401

[27] Hodge, A., Robinson, D. & Fitter, A. Are microorganisms more effective than plants at competing for nitrogen? *Trends Plant Sci.* **5**, 304–308 (2000). 10871903 10.1016/S1360-1385(00)01656-3

[28] Kuzyakov, Y. & Xu, X. Competition between roots and microorganisms for nitrogen: mechanisms and ecological relevance. *N. Phytol.* **198**, 656–669 (2013). 10.1111/nph.12235 23521345

[29] Repo, M. et al. Large N_2_O emissions from cryoturbated peat soil in tundra. *Nat. Geosci.* **2**, 189–192 (2009). 10.1038/ngeo434

[30] Marushchak, M. E. et al. Hot spots for nitrous oxide emissions found in different types of permafrost peatlands. *Glob. Change Biol.* **17**, 2601–2614 (2011). 10.1111/j.1365-2486.2011.02442.x

[31] Abbott, B. W. & Jones, J. B. Permafrost collapse alters soil carbon stocks, respiration, CH_4_, and N_2_O in upland tundra. *Glob. Change Biol.* **21**, 4570–4587 (2015). 10.1111/gcb.13069 26301544

[32] Voigt, C. et al. Warming of subarctic tundra increases emissions of all three important greenhouse gases – carbon dioxide, methane, and nitrous oxide. *Glob. Change Biol.* **23**, 3121–3138 (2017). 10.1111/gcb.13563 27862698

[33] Palmer, K., Biasi, C. & Horn, M. A. Contrasting denitrifier communities relate to contrasting N_2_O emission patterns from acidic peat soils in arctic tundra. *ISME J.* **6**, 1058–1077 (2012). 22134649 10.1038/ismej.2011.172 PMC3329112

[34] Gil, J., Pérez, T., Boering, K., Martikainen, P. J. & Biasi, C. Mechanisms responsible for high N_2_O emissions from subarctic permafrost peatlands studied via stable isotope techniques. *Glob. Biogeochem. Cycles* **31**, 172–189 (2017). 10.1002/2015GB005370

[35] Bernhardt, E. S. et al. Control points in ecosystems: moving beyond the hot spot hot moment concept. *Ecosystems* **20**, 665–682 (2017). 10.1007/s10021-016-0103-y

[36] Ramm et al. A review of the importance of mineral nitrogen cycling in the plant-soil-microbe system of permafrost-affected soils—changing the paradigm. *Environ. Res. Lett.* **17**, 013004 (2022). 10.1088/1748-9326/ac417e

[37] Hinkel, K. M. et al. Spatial extent, age, and carbon stocks in drained Thaw Lake Basins on the Barrow Peninsula, Alaska. *Arct. Antarct. Alp. Res.* **35**, 291–300 (2003). 10.1657/1523-0430(2003)035[0291:SEAACS]2.0.CO;2

[38] Zulueta, R. C., Oechel, W. C., Loescher, H. W., Lawrence, W. T. & Paw U, K. T. Aircraft-derived regional scale CO_2_ fluxes from vegetated drained thaw-lake basins and interstitial tundra on the Arctic Coastal Plain of Alaska. *Glob. Change Biol.* **17**, 2781–2802 (2011). 10.1111/j.1365-2486.2011.02433.x

[39] Lara, M. et al. Tundra landform and vegetation productivity trend maps for the Arctic Coastal Plain of northern Alaska. *Sci. Data* **5**, 180058 (2018). 29633984 10.1038/sdata.2018.58 PMC5892374

[40] Liljedahl, A. et al. Pan-Arctic ice-wedge degradation in warming permafrost and its influence on tundra hydrology. *Nat. Geosci.* **9**, 312–318 (2016). 10.1038/ngeo2674

[41] Minke, M., Donner, N., Karpov, N. S., Seifert, N. & Joosten, H. A window to the soul: biotic/abiotic feedback mechanisms in polygon mire ecosystems. in *Influence of climatic and ecological changes on permafrost ecosystems*. *Proceedings of the third international conference “The role of permafrost ecosystems in global climate change*” (YSC Publishing House SB RAS, 2007).

[42] Tieszen, L. L. *Vegetation and Production Ecology of an Alaskan Arctic Tundra*, (eds Tieszen, L. L.) pp. 3–18, (Springer, 1978).

[43] Davidson, S. J. et al Mapping arctic tundra vegetation communities using field spectroscopy and multispectral satellite data in North Alaska, USA. *Remote Sens.* **8**. 10.3390/rs8120978 (2016).

[44] Jorgenson, M. T., & Shur, Y. Evolution of lakes and basins in Northern Alaska and discussion of the thaw lake cycle. *J. Geophys. Res. Earth Surface* **112**. 10.1029/2006JF000531 (2007).

[45] Sturtevant, C. S. & Oechel, W. C. Spatial variation in landscape-level CO_2_ and CH_4_ fluxes from arctic coastal tundra: influence from vegetation, wetness, and the thaw lake cycle. *Glob. Change Biol.* **19**, 2853–2866 (2013). 10.1111/gcb.12247 23649775

[46] Martin, A. F., Lantz, T. C. & Humphreys, E. R. Ice wedge degradation and CO_2_ and CH_4_ Northwest Territories. *Arct. Sci.* **4**, 130–145 (2018).

[47] Taş, N. et al. Landscape topography structures the soil microbiome in Arctic polygonal Tundra. *Nat. Commun.* **9**. 10.1038/s41467-018-03089-z (2018). 10.1038/s41467-018-03089-z PMC5823929 29472560

[48] McGuire, A. D. et al. An assessment of the carbon balance of Arctic Tundra: comparisons among observations, process models, and atmospheric inversions. *Biogeosciences* **9**, 3185–3204 (2012). 10.5194/bg-9-3185-2012

[49] Euskirchen, E. S., Bret-Harte, M. S., Shaver, G. R., Edgar, C. W., & Romanovsky, V. E. Long-term release of carbon dioxide from Arctic Tundra ecosystems in Alaska. *Ecosystems*10.1007/s10021-016-0085-9 (2017).

[50] Steedman, A. E., Lantz, T. C. & Kokelj, S. V. Spatio-temporal variation in high-centre polygons and ice-wedge melt ponds, Tuktoyaktuk Coastlands, Northwest Territories. *Permaf. Periglac. Process.* **28**, 66–78 (2017). 10.1002/ppp.1880

[51] Jorgenson, M. T., Racine, C. H., Walters, J. C. & Osterkamp, T. E. Permafrost degradation and ecological changes associated with a warming climate in central Alaska. *Clim. Change* **48**, 551–579 (2001). 10.1023/A:1005667424292

[52] Turetsky, M. R. et al. Permafrost collapse is accelerating carbon release. *Nature* **569**, 32–34 (2019). 31040419 10.1038/d41586-019-01313-4

[53] Zuidhoff, F. S. & Kolstrup, E. Palsa development and associated vegetation in northern Sweden. *Arct. Antarct. Alp. Res.* **37**, 49–60 (2005). 10.1657/1523-0430(2005)037[0049:PDAAVI]2.0.CO;2

[54] Olefeldt, D. et al. Circumpolar distribution and carbon storage of thermokarst landscapes. *Nat. Commun.* **7**, 13043 (2016). 27725633 10.1038/ncomms13043 PMC5062615

[55] Wilkerson, J. et al. Permafrost nitrous oxide emissions observed on a landscape scale using the airborne eddy-covariance method. *Atmos. Chem. Phys.* **19**, 4257–4268 (2019). 10.5194/acp-19-4257-2019

[56] Pärn, J. et al. Nitrogen-rich organic soils under warm well-drained conditions are global nitrous oxide emission hotspots. *Nat. Commun.* **9**, 1135 (2018). 29555906 10.1038/s41467-018-03540-1 PMC5859301

[57] Marushchak, M. E. et al. Thawing Yedoma permafrost is a neglected nitrous oxide source. *Nat. Commun.* **12**. 10.1038/s41467-021-27386-2 (2021). 10.1038/s41467-021-27386-2 PMC8651752 34876586

[58] Voigt, C. et al. Increased nitrous oxide emissions from Arctic peatlands after permafrost thaw. *Proc. Natl Acad. Sci. USA* **114**, 6238–6243 (2017). 28559346 10.1073/pnas.1702902114 PMC5474798

[59] Diáková, K. et al. Variation in N_2_ fixation in subarctic tundra in relation to landscape position and nitrogen pools and fluxes. *Arct. Antarct. Alp. Res.* **48**, 111–125 (2016). 10.1657/AAAR0014-064

[60] Stewart, K. J., Lamb, E. G., Coxson, D. S. & Siciliano, S. D. Bryophyte- cyanobacterial associations as a key factor in N_2_-fixation across the Canadian Arctic. *Plant Soil* **344**, 335–346 (2011). 10.1007/s11104-011-0750-x

[61] Arndt, K. A., Lipson, D. A., Hashemi, J., Oechel, W. C. & Zona, D. Snow melt stimulates ecosystem respiration in Arctic ecosystems. *Glob. Change Biol.* **26**, 5042–5051 (2020). 10.1111/gcb.15193 32602589

[62] Wilkman, E. et al. Temperature response of respiration across the heterogeneous landscape of the Alaskan Arctic tundra. *J. Geophys. Res. Biogeosci.* **123**, 2287–2302 (2018). 10.1029/2017JG004227

[63] Altshuler, I. et al. Denitrifiers, nitrogen-fixing bacteria and N_2_O soil gas flux in high Arctic ice-wedge polygon cryosols. *FEMS Microbiol. Ecol.* **95**, fiz049 (2019). 31034011 10.1093/femsec/fiz049

[64] Stewart, K. J., Grogan, P., Coxson, D. S. & Siciliano, S. D. Topography as a key factor driving atmospheric nitrogen exchanges in Arctic terrestrial ecosystems. *Soil Biol. Biochem.* **70**, 96–112 (2014). 10.1016/j.soilbio.2013.12.005

[65] Elberling, B., Christiansen, H. & Hansen, B. High nitrous oxide production from thawing permafrost. *Nat. Geosci.* **3**, 332–335 (2010). 10.1038/ngeo803

[66] Bai, E., Houlton, B. Z. & Wang, Y. P. Isotopic identification of nitrogen hotspots across natural terrestrial ecosystems. *Biogeosciences* **9**, 3287–3304 (2012). 10.5194/bg-9-3287-2012

[67] Lipson, D. A. et al. Water table height and microtopography control biogeochemical cycling in an Arctic coastal tundra ecosystem. *Biogeosci. Discuss.* **8**, 6345–6382 (2011).

[68] Butterbach-Bahl, K. et al. Nitrogen processes in terrestrial ecosystems. *Eur. Nitrogen Assess. Sour. Effects Policy Perspect.* (99–125). 10.1017/CBO9780511976988.009 (2011).

[69] Lipson, D. A. et al. Metagenomic insights into anaerobic metabolism along an Arctic peat soil profile. *PLoS ONE* **8**, e64659 (2013). 23741360 10.1371/journal.pone.0064659 PMC3669403

[70] Lipson, D. A. et al. Changes in microbial communities along redox gradients in polygonized Arctic wet tundra soils. *Environ. Microbiol. Rep.* **7**, 649–657 (2015). 26034016 10.1111/1758-2229.12301

[71] Liimatainen, M. et al. Factors controlling nitrous oxide emissions from managed northern peat soils with low carbon to nitrogen ratio. *Soil Biol. Biochem.* **122**, 186–195 (2018). 10.1016/j.soilbio.2018.04.006

[72] Oelke, C., Zhang, T. & Serreze, M. C. Modeling evidence for recent warming of the Arctic soil thermal regime. *Geophys. Res. Lett.* **31**, L07208 (2004). 10.1029/2003GL019300

[73] Lewkowicz, A. G. & Way, R. G. Extremes of summer climate trigger thousands of thermokarst landslides in a High Arctic environment. *Nat. Commun.* **10**, 1329 (2019). 30940802 10.1038/s41467-019-09314-7 PMC6445831

[74] Fiencke, C., Marushchak, M. E., Sanders, T., Wegner, R. & Beer, C. Microbiogeochemical traits to identify nitrogen hotspots in permafrost regions. *Nitrogen* **3**, 458–501 (2022). 10.3390/nitrogen3030031

[75] Siewert, M. B., Lantuit, H., Richter, A. & Hugelius, G. Permafrost causes unique fine-scale spatial variability across Tundra soils. *Glob. Biogeochem. Cycles* **35**, 1–19 (2021). 10.1029/2020GB006659

[76] Mastepanov, M. et al. Revisiting factors controlling methane emissions from high-Arctic tundra. *Biogeosciences* **10**, 5139–5158 (2013). 10.5194/bg-10-5139-2013

[77] Bockheim, J. G., Hinkel, K. M. & Nelson, F. E. Soils of the barrow region, Alaska1. *Polar Geogr.* **25**, 163–181 (2001). 10.1080/10889370109377711

[78] San Martin Ruiz, M., Reiser, M. & Kranert, M. Nitrous oxide emission fluxes in coffee plantations during fertilization: a case study in Costa Rica. *Atmosphere* **12**, 1656 (2021). 10.3390/atmos12121656

[79] Elpelt-Wessel, I., Reiser, M., Morrison, D. & Kranert, M. Emission determination by three remote sensing methods in two release trials. *Atmosphere* **13**, 53 (2022). 10.3390/atmos13010053

[80] McEwing, K. R., Fisher, J. P. & Zona, D. Environmental and vegetation controls on the spatial variability of CH_4_ emission from wet-sedge and tussock tundra ecosystems in the Arctic. *Plant Soil* **388**, 37–52 (2015). 25834292 10.1007/s11104-014-2377-1 PMC4372828

[81] Ekaso, D., Nex, F. & Kerle, N. Accuracy assessment of real-time kinematics (RTK) measurements on unmanned aerial vehicles (UAV) for direct geo-referencing. *Geo-Spat. Inf. Sci.* **23**, 165–181 (2020). 10.1080/10095020.2019.1710437

[82] Zona, D. et al. Delayed responses of an Arctic ecosystem to an extreme summer: impacts on net ecosystem exchange and vegetation functioning. *Biogeosciences* **11**, 5877–5888 (2014). 10.5194/bg-11-5877-2014

[83] Carter, M. & Gregorich, E. *Soil sampling and methods of analysis*. 2nd edn, (CRC Press, 2008).

[84] Monhonval. A.,Mauclet, E. et al. Mineral organic carbon interactions in dry versus wet tundra soils. *Geoderma* **436**, 116552 (2023). 10.1016/j.geoderma.2023.116552

[85] R Core Team R: A language and environment for statistical computing. R Foundation for Statistical Computing, Vienna, Austria. https://www.R-project.org/ (2020).

[86] Dowle, A. and Srinivasan, A. data.table: Extension of ‘data.frame‘. R package version 1.14.2. https://CRAN.R-project.org/package=data.table (2021).

[87] Hebbali, A. olsrr: Tools for Building OLS Regression Models. R package version 0.5.3. https://CRAN.R-project.org/package=olsrr (2020).

[88] Wickham, H. ggplot2: Elegant Graphics for Data Analysis. Springer-Verlag New York. (2016).

[89] Ahlmann-Eltze, C. & Patil, I. ggsignif: R Package for Displaying Significance Brackets for ‘ggplot2’. PsyArxiv. 10.31234/osf.io/7awm6 (2021).

[90] Wilke, C. cowplot: Streamlined Plot Theme and Plot Annotations for ‘ggplot2’. R package version 1.1.0. https://CRAN.R-project.org/package=cowplot (2020).

[91] Long, J. A. interactions: Comprehensive, User-Friendly Toolkit for Probing Interactions. R package version 1.1.0. https://CRAN.R-project.org/package=interactions (2019).

