# Supplementary material for: Thermokarst landscape exhibits large nitrous oxide emissions in Alaska’s coastal polygonal tundra
Source: Commun Earth Environ. 2024 Aug 30;5(1):473. doi: 10.1038/s43247-024-01583-5 (PMC11364506; doi:10.1038/s43247-024-01583-5)
Supplement: Supplementary file 2 — Supplementary Material [file 43247_2024_1583_MOESM2_ESM.pdf]

Supplementary Material for article "Thermokarst landscape exhibits large nitrous oxide emissions in Alaska's coastal polygonal tundra"

J. Hashemi, D. A. Lipson, K. A. Arndt, S. J. Davidson, A. Kalhori, K. Lunneberg, L. van Delden, W.C. Oechel, & D. Zona

Supplementary Figure 1. Photos of thermokarst polygon surfaces on the Barrow environmental observatory, near Utqiaġvik, AK.

Supplementary Figure 2. Comparisons of soil temperature, soil water content, thaw depth, and bulk density at vegetated and unvegetated areas on thermokarst polygons.

Supplementary Figure 3. UAV Imagery of study site and the distribution of thermokarst polygon landscape features.

Supplementary Figure 4. Partial diurnal trend of N_2O fluxes at vegetated and unvegetated areas on thermokarst polygons.

Supplementary Figure 5. Regression of thaw depth and N_2O fluxes at vegetated and unvegetated areas on thermokarst polygons.

Supplementary Figure 6. Nitrogen (^{15}N) and carbon (^{13}C) stable isotope ratios and variability with soil depth at vegetated and unvegetated areas on thermokarst polygons.

Supplementary Figure 7. Total soil nitrogen content at vegetated and unvegetated areas on thermokarst polygons both raw data and corrected with mean bulk density.

Supplementary Figure 8. Carbon to nitrogen ratios and variability with soil depth at vegetated and unvegetated areas on thermokarst polygons.

Supplementary Figure 9. Comparisons of soil content per volume of N and C and soil depth at vegetated and unvegetated areas on thermokarst polygon features

Supplementary Figure 10. Static flux chamber and Fourier transform infrared greenhouse gas analyzer setup.

Supplementary Table 1. Multivariate regression model performance

Supplementary Figures

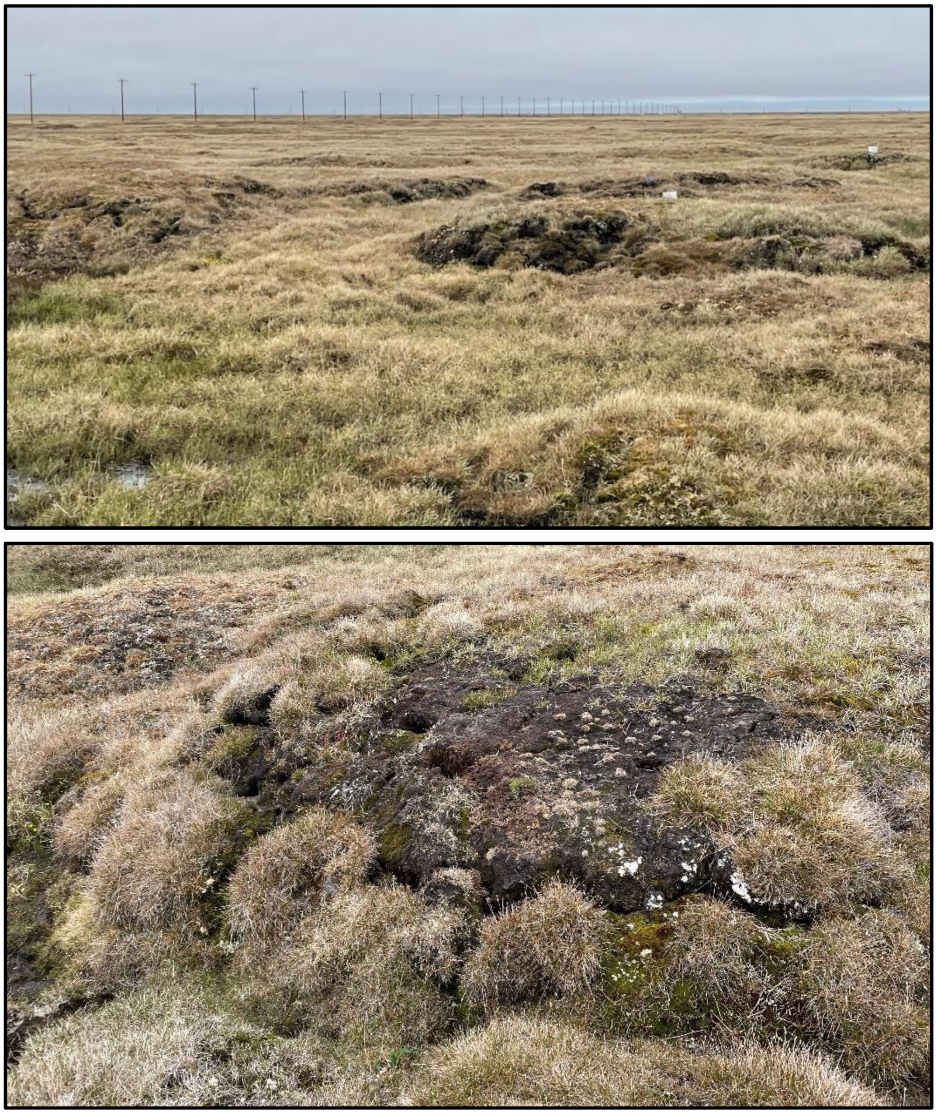

Supplementary Figure 1. Photos of landscape and a thermokarst affected high-centered polygon exhibiting both erosion and vegetation disturbance at the surface.

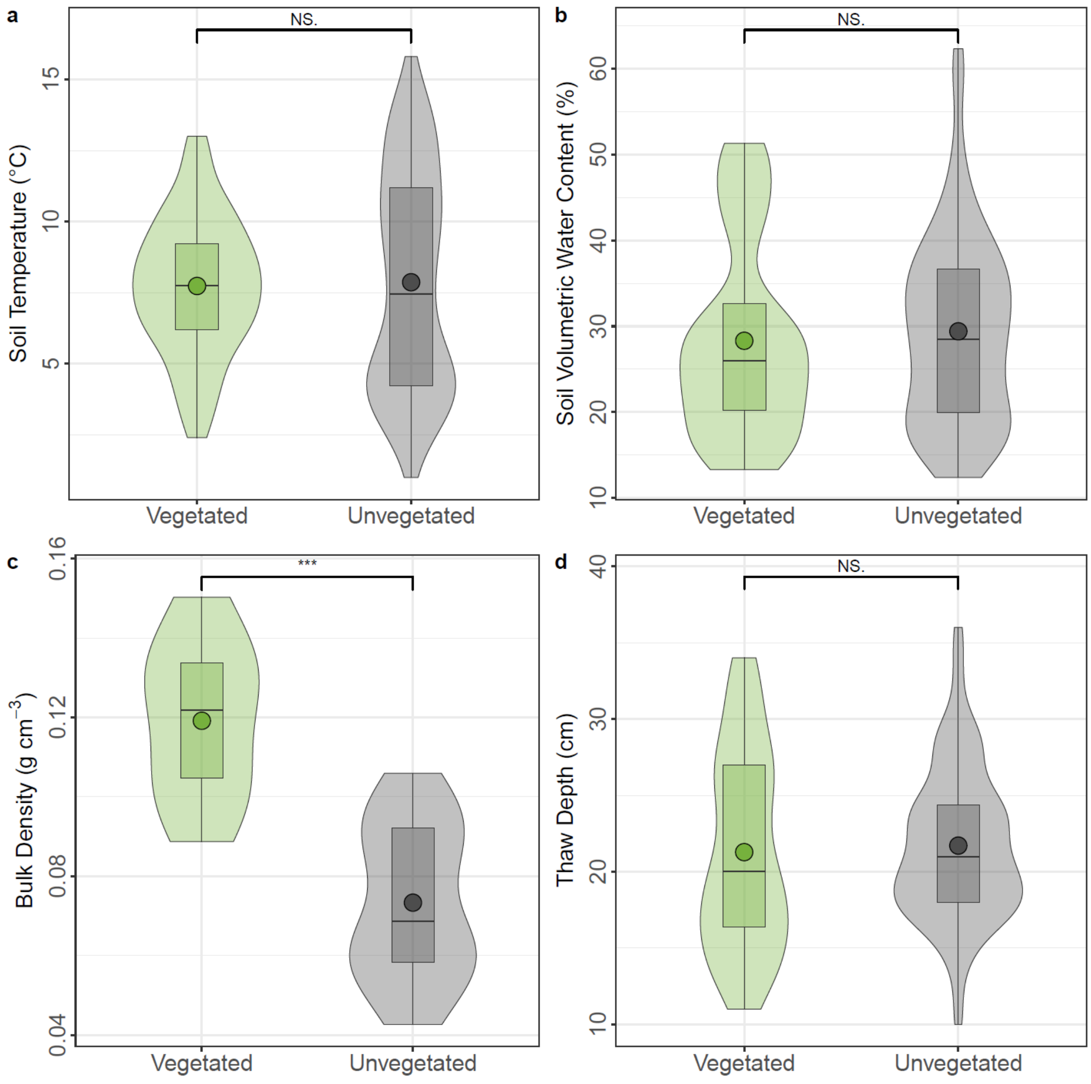

Supplementary Figure 2. Comparisons of soil parameters at vegetated and unvegetated areas on thermokarst polygon features including (a) soil temperature, (b) thaw depth, (c) soil water content (0-15cm) and (d) bulk density (0-15cm). *** = p<0.001 and NS. indicates no significance.

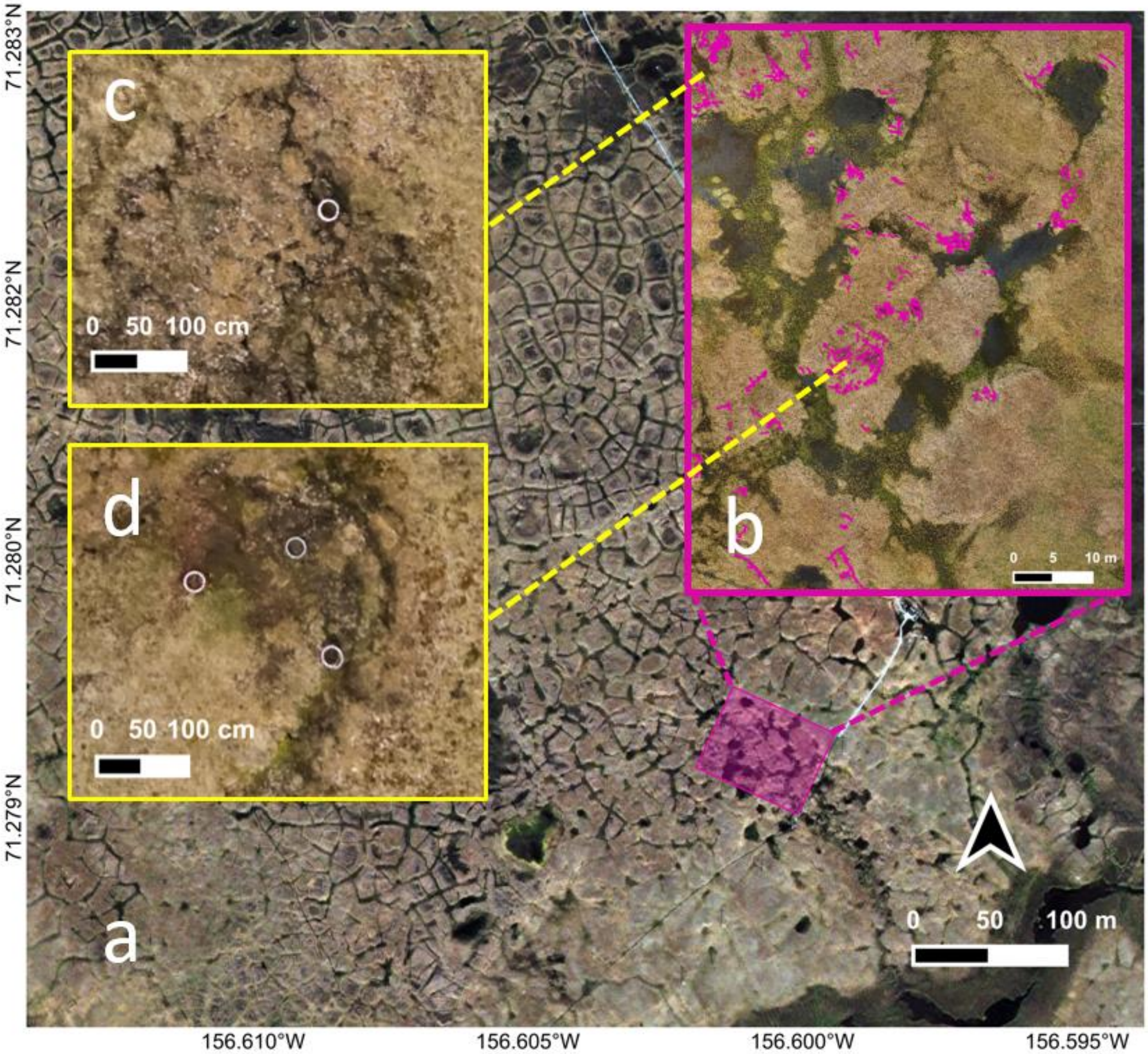

Supplementary Figure 3. Nested imagery of (a) polygonized tundra around study site; (b) UAV imagery with spatial resolution of ~1 cm. Pink areas highlight unvegetated features, comprising about 1.5% of map extent; (b & c) close-up of UAV imagery on some of the unvegetated regions. White circles in panels c & d are sampling collars. UAV imagery was collected using a DJIP4 Multispectral drone flown at 12m above-ground. Imagery Map (a) source credits: Esri, Maxar, GeoEye, Earthstar Geographics, CNES/Airbus DS, USDA, USGS, AeroGRID, IGN and the GIS User Community.

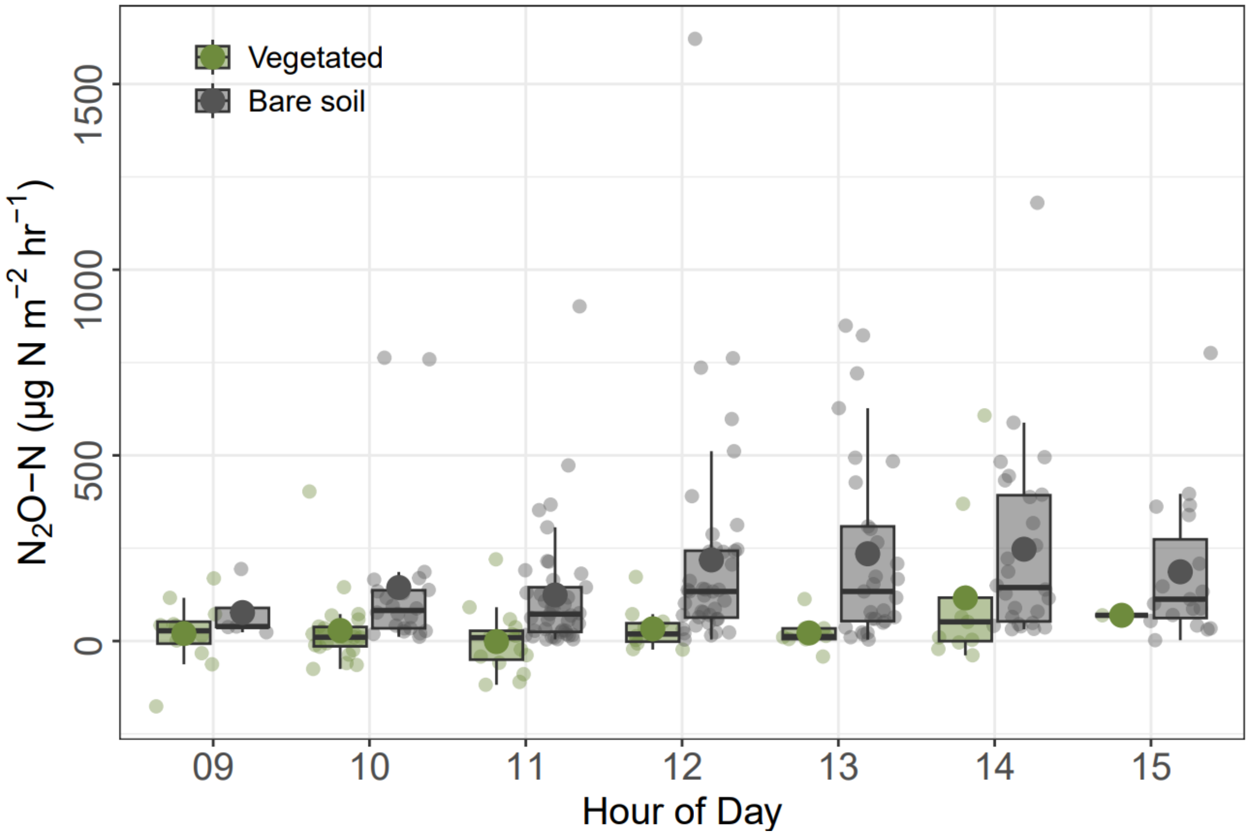

Supplementary Figure 4. N_2O flux and hour of day at vegetated and unvegetated areas on thermokarst polygon features.

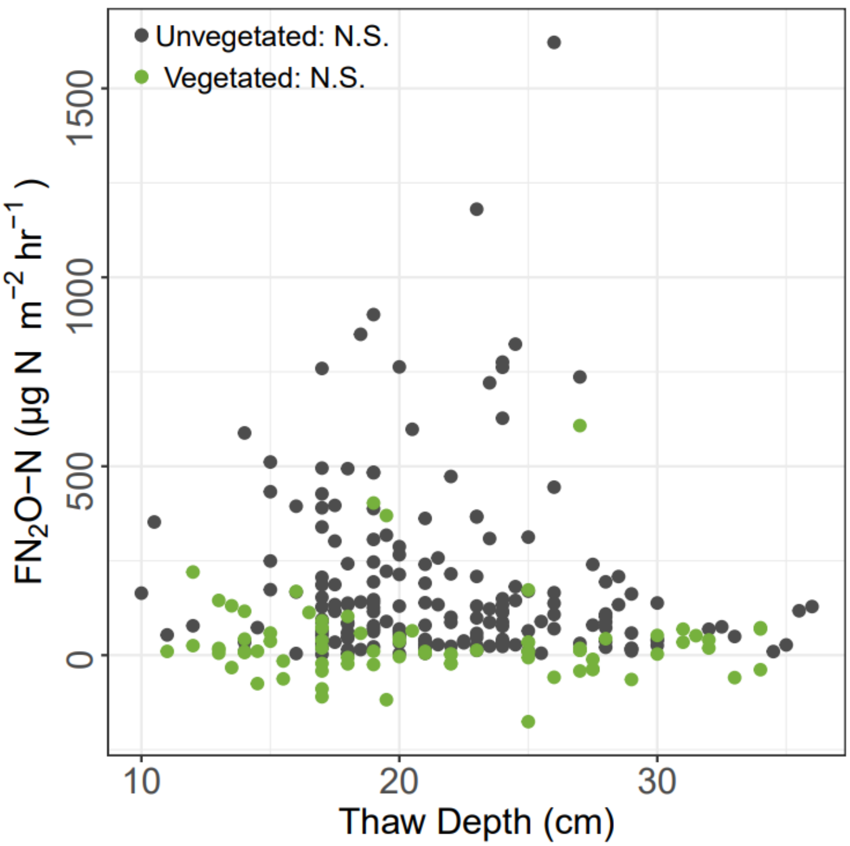

Supplementary Figure 5. N_2O flux and thaw depth at vegetated and unvegetated areas on thermokarst polygon features. NS. Indicates not significant.

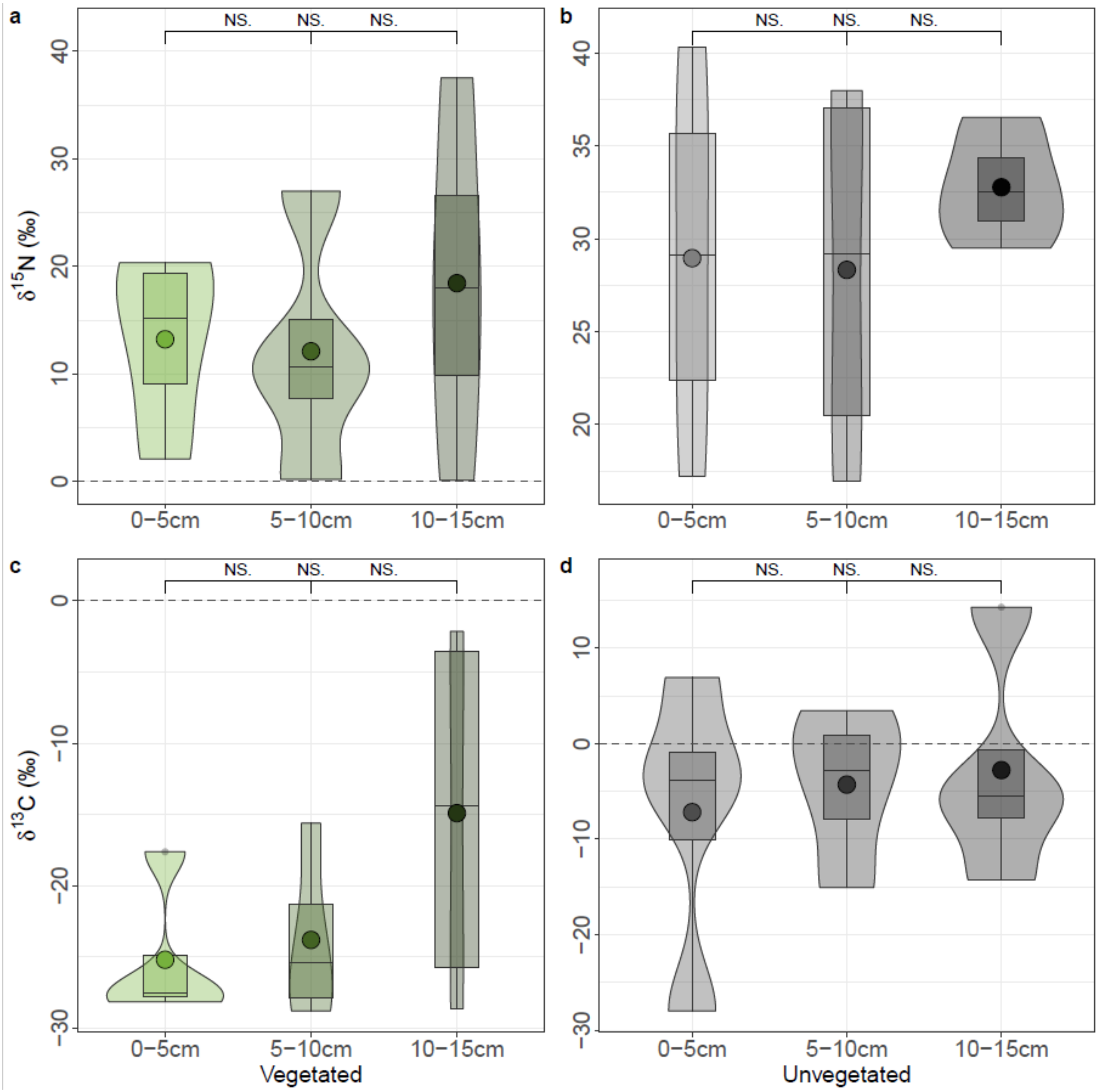

Supplementary Figure 6. Comparisons of stable isotope ratios of d^{15}N (a & b) and d^{13}C (c & d) and soil depth at vegetated and unvegetated areas on thermokarst polygon features. NS. indicates no significance.

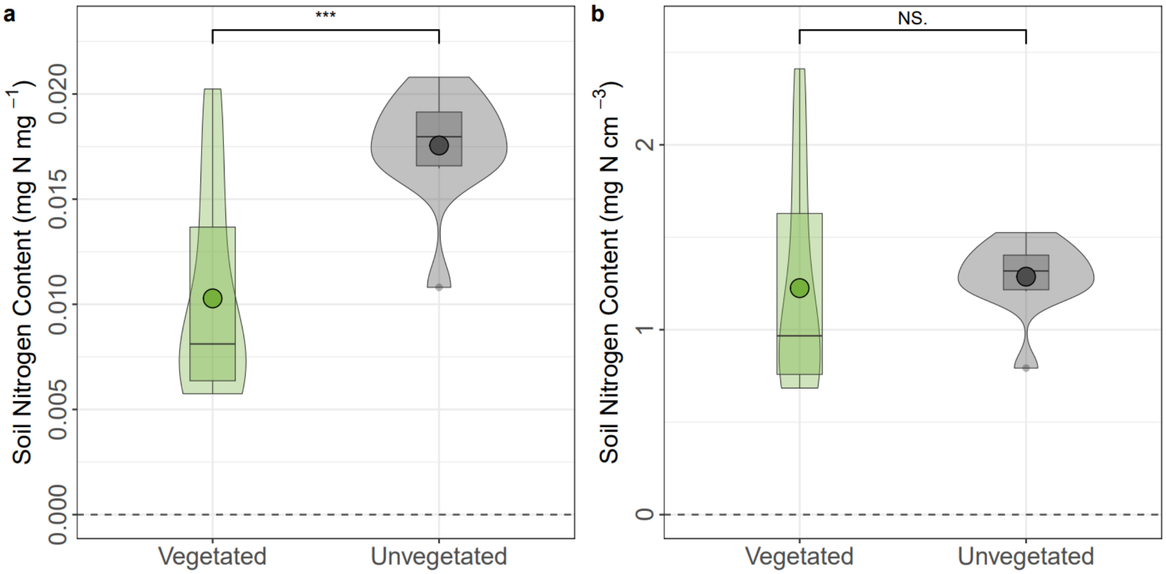

Supplementary Figure 7. Comparisons of soil nitrogen content for 0-15cm of the soil column by (a) soil dry weight and (b) volume at vegetated and unvegetated areas on thermokarst polygon features. NS. indicates no significance.

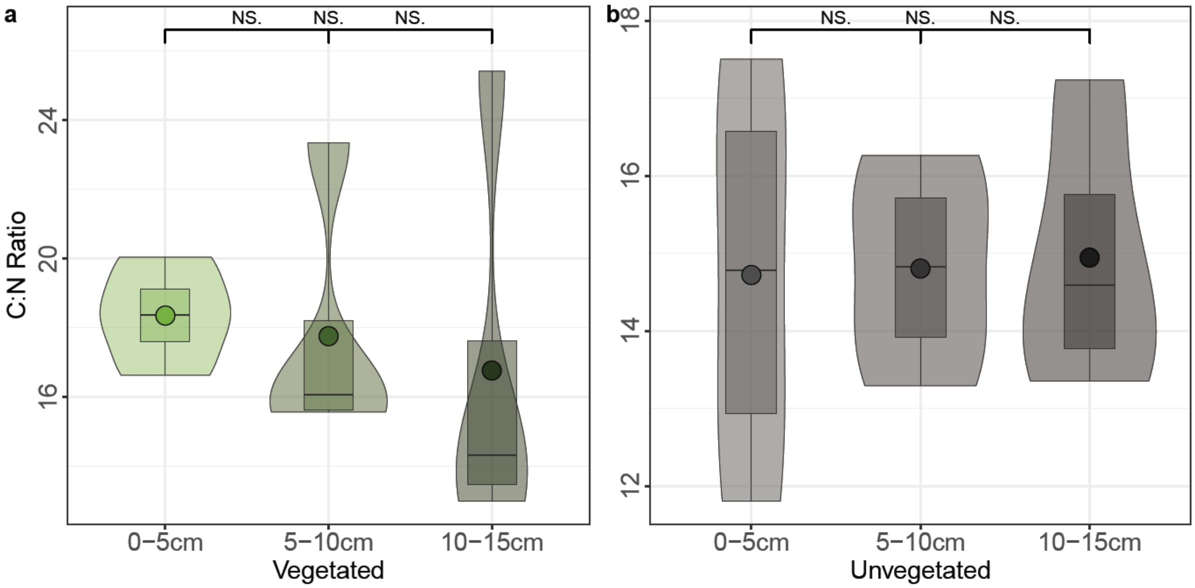

Supplementary Figure 8. Comparisons of carbon to nitrogen ratios and soil depth at (a) unvegetated and (b) vegetated areas on thermokarst polygon features. NS. indicates no significance.

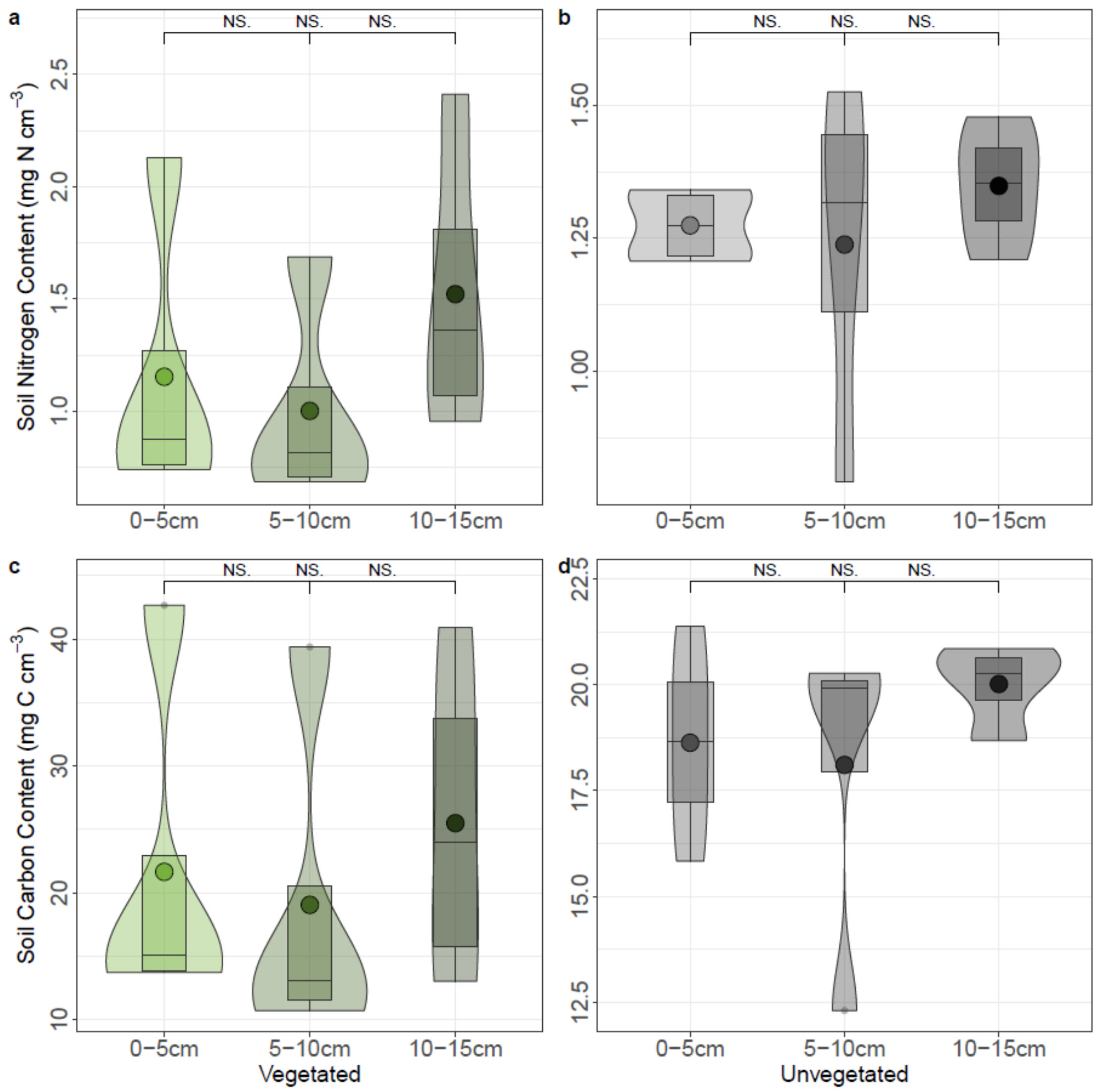

Supplementary Figure 9. Comparisons of soil content per volume of N (a & b) and C (c & d) and soil depth at vegetated and unvegetated areas on thermokarst polygon features. NS. indicates no significance.

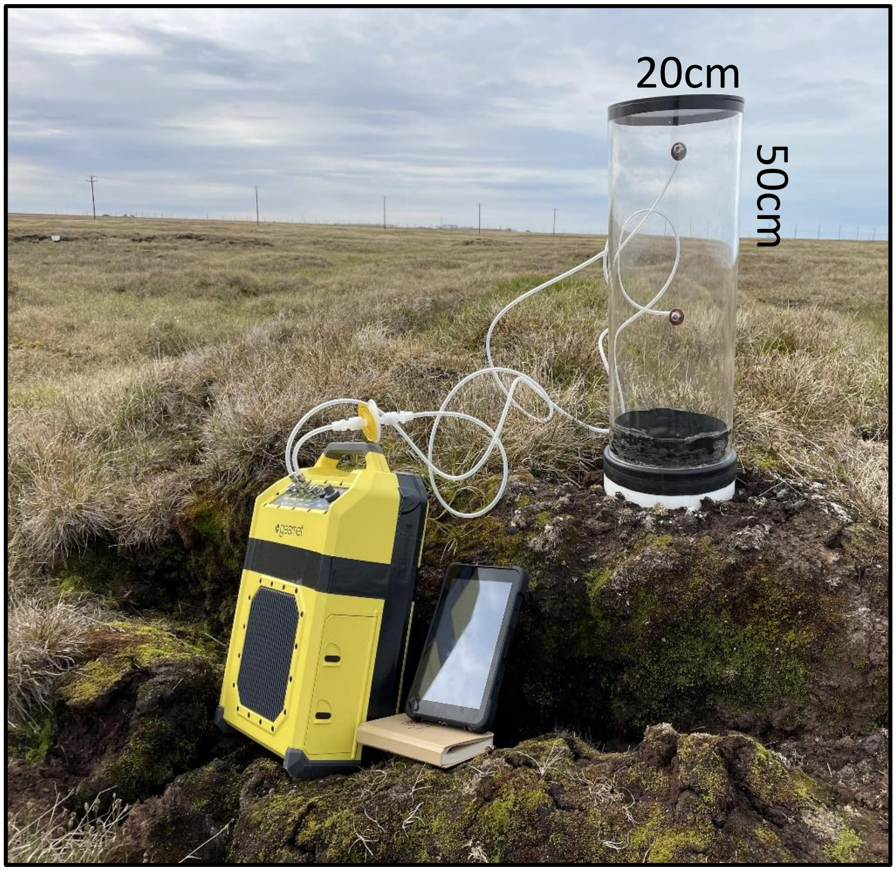

Supplementary Figure 10. Photo of FTIR GGA and chamber setup with dimensions on thermokarst high centered polygon feature.

Supplementary Tables

Supplementary Table 1. Multivariate mixed effects model performance of N2O fluxes at unvegetated areas. Fluxes have been square root transformed, with collar location serving as a random effect to address pseudoreplication.

<table>
<tr><th colspan="8">Model 1 | AIC: 1143.16 | Pseudo-R² (fixed effects) = 0.34 | Pseudo-R² (total) = 0.47</th></tr>
<tr><th></th><th>Coefficients</th><th>CI (lwr)</th><th>CI (upr)</th><th>Std. Error</th><th>DF</th><th>tvalue</th><th>pvalue</th></tr>
<tr><td>Intercept</td><td>15.10196808</td><td>8.762812466</td><td>21.45922947</td><td>3.258855683</td><td>177.1218345</td><td>4.634132207</td><td>6.93E-06</td></tr>
<tr><td>ST</td><td>0.828003164</td><td>0.17533174</td><td>1.486102014</td><td>0.335352207</td><td>169.8009669</td><td>2.469055363</td><td>0.014536498</td></tr>
<tr><td>ST:WFPS</td><td>-0.024227205</td><td>-0.045164719</td><td>-0.003476889</td><td>0.01066515</td><td>170.7088162</td><td>-2.271623346</td><td>0.02435765</td></tr>
<tr><td>WFPS</td><td>-0.151036739</td><td>-0.336997196</td><td>0.034258334</td><td>0.095204918</td><td>177.6256169</td><td>-1.586438409</td><td>0.114418483</td></tr>
<tr><th colspan="8">Model 2 | AIC: 1145.13 | Pseudo-R² (fixed effects) = 0.34 | Pseudo-R² (total) = 0.48</th></tr>
<tr><th></th><th>Coefficients</th><th>CI (lwr)</th><th>CI (upr)</th><th>Std. Error</th><th>DF</th><th>tvalue</th><th>pvalue</th></tr>
<tr><td>Intercept</td><td>15.49677126</td><td>7.766236372</td><td>23.23440097</td><td>3.990314228</td><td>164.364502</td><td>3.883596722</td><td>0.00014887</td></tr>
<tr><td>ST:WFPS</td><td>-0.024430473</td><td>-0.04549944</td><td>-0.003551247</td><td>0.0107681</td><td>173.7383113</td><td>-2.268782086</td><td>0.024511764</td></tr>
<tr><td>ST</td><td>0.828088525</td><td>0.175931261</td><td>1.48642275</td><td>0.336092179</td><td>169.4140233</td><td>2.463873235</td><td>0.014743107</td></tr>
<tr><td>TD</td><td>-0.01989239</td><td>-0.240759956</td><td>0.201034704</td><td>0.114280772</td><td>91.91929824</td><td>-0.174065943</td><td>0.862196427</td></tr>
<tr><td>WFPS</td><td>-0.148325279</td><td>-0.336612344</td><td>0.038909489</td><td>0.096610313</td><td>176.9539182</td><td>-1.535294478</td><td>0.126497561</td></tr>
<tr><th colspan="8">Model 3 | AIC: 1146.37 | Pseudo-R² (fixed effects) = 0.32 | Pseudo-R² (total) = 0.46</th></tr>
<tr><th></th><th>Coefficients</th><th>CI (lwr)</th><th>CI (upr)</th><th>Std. Error</th><th>DF</th><th>tvalue</th><th>pvalue</th></tr>
<tr><td>Intercept</td><td>21.09549494</td><td>17.32422038</td><td>24.88391731</td><td>1.932229493</td><td>133.5168631</td><td>10.91769638</td><td>3.16E-20</td></tr>
<tr><td>ST</td><td>0.105278775</td><td>-0.104806427</td><td>0.315014945</td><td>0.107096589</td><td>164.7059483</td><td>0.983026412</td><td>0.327036236</td></tr>
<tr><td>WFPS</td><td>-0.34184139</td><td>-0.430874225</td><td>-0.253605951</td><td>0.045148022</td><td>147.5574748</td><td>-7.571569618</td><td>3.71E-12</td></tr>
<tr><th colspan="8">Model 4 | AIC: 1148.36 | Pseudo-R² (fixed effects) = 0.32 | Pseudo-R² (total) = 0.46</th></tr>
<tr><th></th><th>Coefficients</th><th>CI (lwr)</th><th>CI (upr)</th><th>Std. Error</th><th>DF</th><th>tvalue</th><th>pvalue</th></tr>
<tr><td>Intercept</td><td>20.81222792</td><td>14.45657862</td><td>27.18945466</td><td>3.269999341</td><td>99.21116807</td><td>6.364596978</td><td>6.10E-09</td></tr>
<tr><td>ST</td><td>0.108945436</td><td>-0.111979437</td><td>0.328548786</td><td>0.112811607</td><td>174.6633332</td><td>0.965728963</td><td>0.335515054</td></tr>
<tr><td>TD</td><td>0.01197467</td><td>-0.211427433</td><td>0.234953891</td><td>0.115018132</td><td>100.2672471</td><td>0.104111152</td><td>0.917289107</td></tr>
<tr><td>WFPS</td><td>-0.342025563</td><td>-0.431741653</td><td>-0.253632118</td><td>0.045560522</td><td>149.7281179</td><td>-7.507059793</td><td>5.02E-12</td></tr>
<tr><th colspan="8">Model 5 | AIC: 1149.73 | Pseudo-R² (fixed effects) = 0.32 | Pseudo-R² (total) = 0.46</th></tr>
<tr><th></th><th>Coefficients</th><th>CI (lwr)</th><th>CI (upr)</th><th>Std. Error</th><th>DF</th><th>tvalue</th><th>pvalue</th></tr>
<tr><td>Intercept</td><td>15.84502692</td><td>1.635713579</td><td>29.89519834</td><td>7.258829215</td><td>176.8102127</td><td>2.182862614</td><td>0.030363959</td></tr>
<tr><td>ST</td><td>0.105647696</td><td>-0.115012331</td><td>0.325217668</td><td>0.113075147</td><td>174.0587345</td><td>0.934314031</td><td>0.351436271</td></tr>
<tr><td>TD</td><td>0.259475891</td><td>-0.401672107</td><td>0.933231976</td><td>0.341968827</td><td>175.5260066</td><td>0.758770596</td><td>0.449007481</td></tr>
<tr><td>WFPS:TD</td><td>-0.007874199</td><td>-0.02808588</td><td>0.011891247</td><td>0.010229329</td><td>176.4463602</td><td>-0.769766922</td><td>0.442467472</td></tr>
<tr><td>WFPS</td><td>-0.180790846</td><td>-0.596284836</td><td>0.241422912</td><td>0.214866537</td><td>176.5423196</td><td>-0.841409965</td><td>0.401256574</td></tr>
<tr><th colspan="8">Model 6 | AIC: 1150.3 | Pseudo-R² (fixed effects) = 0.32 | Pseudo-R² (total) = 0.46</th></tr>
<tr><th></th><th>Coefficients</th><th>CI (lwr)</th><th>CI (upr)</th><th>Std. Error</th><th>DF</th><th>tvalue</th><th>pvalue</th></tr>
<tr><td>Intercept</td><td>19.92308746</td><td>10.69336478</td><td>29.26179852</td><td>4.773163166</td><td>172.0595163</td><td>4.173979973</td><td>4.74E-05</td></tr>
<tr><td>ST:TD</td><td>-0.005432062</td><td>-0.046553105</td><td>0.035758985</td><td>0.021106263</td><td>163.3237013</td><td>-0.257367308</td><td>0.797219206</td></tr>
<tr><td>ST</td><td>0.227743586</td><td>-0.700813761</td><td>1.15340558</td><td>0.475384232</td><td>162.6824658</td><td>0.479072656</td><td>0.632530263</td></tr>
<tr><td>TD</td><td>0.050701672</td><td>-0.319569223</td><td>0.418359296</td><td>0.189788832</td><td>176.9223538</td><td>0.267147817</td><td>0.789666498</td></tr>
<tr><td>WFPS</td><td>-0.341444316</td><td>-0.431214643</td><td>-0.252839619</td><td>0.045736908</td><td>148.8825212</td><td>-7.465400137</td><td>6.46E-12</td></tr>
<tr><th colspan="8">Model 7 | AIC: 1194.76 | Pseudo-R² (fixed effects) = 0.03 | Pseudo-R² (total) = 0.36</th></tr>
<tr><th></th><th>Coefficients</th><th>CI (lwr)</th><th>CI (upr)</th><th>Std. Error</th><th>DF</th><th>tvalue</th><th>pvalue</th></tr>
<tr><td>Intercept</td><td>10.38792951</td><td>3.373849738</td><td>17.41664799</td><td>3.597418069</td><td>121.3940424</td><td>2.887606975</td><td>0.00459729</td></tr>
<tr><td>ST</td><td>0.317137232</td><td>0.077493209</td><td>0.557857243</td><td>0.122650196</td><td>172.6996015</td><td>2.585705058</td><td>0.010542824</td></tr>
<tr><td>TD</td><td>-0.055457165</td><td>-0.324385591</td><td>0.212019872</td><td>0.137124944</td><td>137.7065527</td><td>-0.404427987</td><td>0.686525482</td></tr>
<tr><th colspan="8">Model 8 | AIC: 1196.41 | Pseudo-R² (fixed effects) = 0.03 | Pseudo-R² (total) = 0.36</th></tr>
<tr><th></th><th>Coefficients</th><th>CI (lwr)</th><th>CI (upr)</th><th>Std. Error</th><th>DF</th><th>tvalue</th><th>pvalue</th></tr>
<tr><td>Intercept</td><td>8.21225268</td><td>-1.852079466</td><td>18.32665114</td><td>5.177336026</td><td>176.7939693</td><td>1.586192713</td><td>0.114482571</td></tr>
<tr><td>ST:TD</td><td>-0.013737501</td><td>-0.059600709</td><td>0.032015553</td><td>0.023436909</td><td>162.4589278</td><td>-0.586148174</td><td>0.558589426</td></tr>
<tr><td>ST</td><td>0.616312389</td><td>-0.408013851</td><td>1.644299492</td><td>0.524924636</td><td>161.6798207</td><td>1.174096903</td><td>0.242082732</td></tr>
<tr><td>TD</td><td>0.041608857</td><td>-0.380413557</td><td>0.46040111</td><td>0.215407985</td><td>177.4110032</td><td>0.193163022</td><td>0.847052257</td></tr>
</table>
