# Supplementary material for: Thermokarst landscape exhibits large nitrous oxide emissions in Alaska’s coastal polygonal tundra
Source: Commun Earth Environ. 2024 Aug 30;5(1):473. doi: 10.1038/s43247-024-01583-5 (PMC11364506; doi:10.1038/s43247-024-01583-5)
Supplement: Supplementary file 3 — Reporting Summary [file 43247_2024_1583_MOESM3_ESM.pdf]

nature portfolio

Corresponding author(s): Joshua Hashemi

Last updated by author(s): 2024/07/25

Reporting Summary

Nature Portfolio wishes to improve the reproducibility of the work that we publish. This form provides structure for consistency and transparency in reporting. For further information on Nature Portfolio policies, see our Editorial Policies and the Editorial Policy Checklist.

Statistics

For all statistical analyses, confirm that the following items are present in the figure legend, table legend, main text, or Methods section.

n/a	Confirmed	
☐	☒	The exact sample size (*n*) for each experimental group/condition, given as a discrete number and unit of measurement
☐	☒	A statement on whether measurements were taken from distinct samples or whether the same sample was measured repeatedly
☐	☒	The statistical test(s) used AND whether they are one- or two-sided<br>*Only common tests should be described solely by name; describe more complex techniques in the Methods section.*
☐	☒	A description of all covariates tested
☐	☒	A description of any assumptions or corrections, such as tests of normality and adjustment for multiple comparisons
☐	☒	A full description of the statistical parameters including central tendency (e.g. means) or other basic estimates (e.g. regression coefficient) AND variation (e.g. standard deviation) or associated estimates of uncertainty (e.g. confidence intervals)
☐	☒	For null hypothesis testing, the test statistic (e.g. *F*, *t*, *r*) with confidence intervals, effect sizes, degrees of freedom and *P* value noted<br>*Give P values as exact values whenever suitable.*
☒	☐	For Bayesian analysis, information on the choice of priors and Markov chain Monte Carlo settings
☒	☐	For hierarchical and complex designs, identification of the appropriate level for tests and full reporting of outcomes
☒	☐	Estimates of effect sizes (e.g. Cohen's *d*, Pearson's *r*), indicating how they were calculated

Our web collection on statistics for biologists contains articles on many of the points above.

Software and code

Policy information about availability of computer code

Data collection	The data collection steps used to process the data in this study are explained in detail in the Methods section of the article and in the repository where the data (DOI: 10.5281/zenodo.8391857) are located.
Data analysis	The data description to process and analyze the data in this study are explained in detail in the Methods section of the article, Supplementary Table 1, and in the repository where the data (DOI: 10.5281/zenodo.8391857) are located.

For manuscripts utilizing custom algorithms or software that are central to the research but not yet described in published literature, software must be made available to editors and reviewers. We strongly encourage code deposition in a community repository (e.g. GitHub). See the Nature Portfolio guidelines for submitting code & software for further information.

Data

Policy information about availability of data

All manuscripts must include a data availability statement. This statement should provide the following information, where applicable:

- Accession codes, unique identifiers, or web links for publicly available datasets
- A description of any restrictions on data availability
- For clinical datasets or third party data, please ensure that the statement adheres to our policy

All data that support the findings of this study are openly available at: https://doi.org/10.5281/zenodo.8391857

Human research participants

Policy information about studies involving human research participants and Sex and Gender in Research.

Reporting on sex and gender	*Use the terms sex (biological attribute) and gender (shaped by social and cultural circumstances) carefully in order to avoid confusing both terms. Indicate if findings apply to only one sex or gender; describe whether sex and gender were considered in study design whether sex and/or gender was determined based on self-reporting or assigned and methods used. Provide in the source data disaggregated sex and gender data where this information has been collected, and consent has been obtained for sharing of individual-level data; provide overall numbers in this Reporting Summary. Please state if this information has not been collected. Report sex- and gender-based analyses where performed, justify reasons for lack of sex- and gender-based analysis.*
Population characteristics	*Describe the covariate-relevant population characteristics of the human research participants (e.g. age, genotypic information, past and current diagnosis and treatment categories). If you filled out the behavioural & social sciences study design questions and have nothing to add here, write "See above."*
Recruitment	*Describe how participants were recruited. Outline any potential self-selection bias or other biases that may be present and how these are likely to impact results.*
Ethics oversight	*Identify the organization(s) that approved the study protocol.*

Note that full information on the approval of the study protocol must also be provided in the manuscript.

Field-specific reporting

Please select the one below that is the best fit for your research. If you are not sure, read the appropriate sections before making your selection.

☐ Life sciences ☐ Behavioural & social sciences ☒ Ecological, evolutionary & environmental sciences

For a reference copy of the document with all sections, see nature.com/documents/nr-reporting-summary-flat.pdf

Ecological, evolutionary & environmental sciences study design

All studies must disclose on these points even when the disclosure is negative.

Study description	Chamber based fluxes of nitrous oxide and carbon dioxide (net ecosystem exchange) from a thermokarst landscape near Utgiagvik, Alaska on the Arctic Coastal Plain. We outline the magnitude and some of the dominant factors controlling variability in emissions for these thermokarst landscape features. We show that emissions during the growing season from unvegetated high centered polygons (median (mean)=104.7 (187.7) µg N2O-N m-2 hr-1) are substantially higher than mean rates associated with Arctic tundra wetlands, identifying an Arctic nitrous oxide source.
Research sample	Flux measurements focused on areas with thermokarst disturbance resulting in unvegetated soil surfaces. These features were chosen as we thought that the unvegeated surfaces may exhibit differing emission dynamics than surrounding, vegetated areas because of the competition that occurs between microbial consortia and plants for available inorganic nitrogen.
Sampling strategy	No sample-size calculation was performed. Pathways of nitrous oxide production, consumption and emissions are complex and as such, we sampled as many sites and and flux measurements as possible (263 flux measurements at 30 sites) considering limitations in travel time between sites and the duration of the growing season field campaign.
Data collection	Static chamber fluxes were measured with a Gasmet GT5000 Terra Fourier transform infrared (FTIR) GHG analyzer and a clear, cylindrical polycarbonate chamber. Thaw depth, soil water content, and soil temperature were taken at the time of each flux measurement. Bulk density and soil content (d13C, d15N, Total C, Total N) were taken once, at the end of the study. GHG flux data were recorded with the Gasmet GT5000 Terra. Thaw depth, soil water content, soil temperature, and bulk density were recorded manually. Soil content data was measured/recorded with a continuous flow isotope ratio mass spectrometer (IRMS, Delta V Advantage, Thermo Fisher Scientific). All data were collected and recorded by J. Hashemi.
Timing and spatial scale	Measurements were taken as often as possible, whenever weather permitted during the month of July, 2021. Measurements were over a small area (roughly 100x100m) of the tundra where extensive thermokarst disturbance was found.
Data exclusions	No data were excluded from this analysis.
Reproducibility	Our study did not involve controlled experiments.
Randomization	There was no random sampling involved in this study and we did not allocate samples into groups.
Blinding	Blinding was not relevant in our study because there was no allocation form any individuals involved.

Did the study involve field work? ☒ Yes ☐ No

Field work, collection and transport

Field conditions	A polygonal tundra in the continuous permafrost zone. Soils are gelisols (turbels (cryoturbated soils: 71-77%; orthels (mineral): 8%; organic soils: 1%) with high levels total organic C and N. Vegetation primarily consists of wet sedges (Carex aquatilis) and mosses (Sphagnum spp. and Drepanocladus spp.) in heavily inundated areas such as low center polygons and troughs, and moss/lichen (Polytrichum spp. & Dicranum spp.) dominated communities in high-center polygon and ridge areas. The water table is variable depending on landscape relief and can be as high as >20 cm above the ground surface and as low as >50 cm below the ground surface. Collar locations were only in thermokarst polygons with unvegetated soil or adjacent vegetated areas also on thermokarst polygons. All thermokarst polygon features had a water table at or below the active layer depth throughout the study period. Mean maximum active layer thaw depth in these features was estimated at ~40 cm.
Location	This study was conducted near Utqiaġvik, Alaska in a well-developed polygonal tundra consisting of high and low-center polygons, on the Barrow Ecological Observatory (BEO; 71 16' 51''N, 156 26' 44''W)
Access & import/export	The land where the study was conducted is owned by the Ukpeaġvik Inupiat Corporation. The Ukpeaġvik Inupiat Corporation allowed us to access the site via boardwalks from the road into the tundra in an effort to minimize the land disturbance.
Disturbance	Environmental disturbance was minimal for our study. Gas flux and ancillary measurements were taken by probing the soil with flux chamber collars, soil water content sensors, and thaw depth sensors. Soil samples taken for measurements of soil content and bulk density were as small as possible (10x10x15cm). We minimized the walking to study areas as much as possible.

Reporting for specific materials, systems and methods

We require information from authors about some types of materials, experimental systems and methods used in many studies. Here, indicate whether each material, system or method listed is relevant to your study. If you are not sure if a list item applies to your research, read the appropriate section before selecting a response.

Materials & experimental systems

n/a	Involved in the study	
☒	☐	Antibodies
☒	☐	Eukaryotic cell lines
☒	☐	Palaeontology and archaeology
☒	☐	Animals and other organisms
☒	☐	Clinical data
☒	☐	Dual use research of concern

Methods

n/a	Involved in the study	
☒	☐	ChIP-seq
☒	☐	Flow cytometry
☒	☐	MRI-based neuroimaging
